# Explaining Person-by-Item Responses using Person- and Item-Level Predictors via Random Forests and Interpretable Machine Learning in Explanatory Item Response Models

**DOI:** 10.1017/psy.2025.10032

**Published:** 2025-07-31

**Authors:** Sun-Joo Cho, Goodwin Amanda, Jorge Salas, Sophia Mueller

**Affiliations:** Vanderbilt University’s Peabody College, United States

**Keywords:** explanatory item response theory, interpretable machine learning, mixed-effects machine learning, random forests

## Abstract

This study incorporates a random forest (RF) approach to probe complex interactions and nonlinearity among predictors into an item response model with the goal of using a hybrid approach to outperform either an RF or explanatory item response model (EIRM) only in explaining item responses. In the specified model, called EIRM-RF, predicted values using RF are added as a predictor in EIRM to model the nonlinear and interaction effects of person- and item-level predictors in person-by-item response data, while accounting for random effects over persons and items. The results of the EIRM-RF are probed with interpretable machine learning (ML) methods, including feature importance measures, partial dependence plots, accumulated local effect plots, and the *H*-statistic. The EIRM-RF and the interpretable methods are illustrated using an empirical data set to explain differences in reading comprehension in digital versus paper mediums, and the results of EIRM-RF are compared with those of EIRM and RF to show empirical differences in modeling the effects of predictors and random effects among EIRM, RF, and EIRM-RF. In addition, simulation studies are conducted to compare model accuracy among the three models and to evaluate the performance of interpretable ML methods.

## 1 Introduction

### 1.1 Study motivation

This study is motivated by an empirical investigation into which person- and item-level predictors (features)^1^ are important in explaining person-by-item binary responses, rather than focusing on developing predictive models for unseen data or generalizing findings to other similar study designs, unless methods like data splitting and external validation are carefully applied. In the motivating empirical study, the goal is to better understand differences in reading comprehension in digital versus paper mediums by exploring the reading behaviors that are related to them. The explanatory investigation enhances understanding of test-taking behaviors. In addition, it enriches theoretical understanding of test data by providing empirical evidence supporting or challenging existing theories related to important predictors. For example, the theory of New Literacies (Leu et al., 2013) indicates that digital reading overlaps with traditional paper reading, such that readers similarly must turn the written code into understandable language by decoding and accessing the meaning of the words in the text. However, digital reading is unique in important ways because it involves differences like how digital highlights tend to be assessed by grabbing text and then pushing a highlighting button versus holding a highlighter in one’s hand. The theory of New Literacies argues that research must unravel these similarities and differences in order to better understand reading in the 21st century. Methods like those explored in the current study help illustrate the different reading behaviors, like differences in highlighting, use of the dictionary, annotation, response time, and looking back that contribute to comprehension when answering questions in the different mediums.

There are three challenges in the explanatory investigation. First, in statistical modeling, the relationship between predictors and outcomes can take various forms – linear, non-linear, logarithmic, exponential, etc. Determining the most appropriate form of this relationship is crucial for model adequacy^2^ and interpretation. However, in many cases, the true nature of these relationships is not immediately apparent. Second, person- and item-level predictors may interact in complex ways, influencing item responses. These interactions can be challenging to model and interpret, especially when they are non-additive or involve higher-order terms. Third, identifying the most relevant predictors from a large number of predictors is crucial for effective model performance in explaining variability in item responses. The challenge lies in selecting predictors that contribute significantly to the model without introducing redundancy or overfitting.

### 1.2 Related work and limitations of current methods

An explanatory item response model (EIRM; De Boeck & Wilson, 2004) has been applied widely to explain or predict person-by-item responses by using person- and/or item-level predictors (see applications in Baayen et al., 2008; Steacy, 2020). In EIRM as a parametrically structured model, the estimated effects of the predictors (estimated regression coefficients) can be interpreted with statistical inference, allowing for random effects over persons and items. However, in most EIRM applications, the linear effects of the predictors on the logit or probit transformation of item response probabilities are often assumed, without probing for non-linear effects (e.g., Baayen et al., 2008; De Boeck & Wilson, 2004; Steacy, 2020). For the non-linear effects, one needs to manually specify which predictors should be nonlinear and in what way (e.g., quadratic or cubic). Furthermore, while incorporating all possible interaction effects in EIRM offers a comprehensive approach, it poses significant challenges in terms of feasibility, complexity, and model interpretability. In contrast, neglecting these interactions can lead to concerns about model adequacy and completeness of the model’s conclusions. Balancing these considerations is a key aspect of model specifications and applications of EIRMs. In addition, when there are many person- and item-level predictors, it is challenging to select the optimal sets of predictors with which variability in item responses is maximally explained.

Machine learning (ML) methods are capable of modeling highly complex and nonlinear relationships between predictors and outcomes (e.g., Kuhn & Johnson, 2018). Among the ML methods, ensemble approaches, which use multiple models, such as RFs (RF; Breiman, 2001) and gradient boosting (GB; Friedman, 2001), have been employed to enhance prediction accuracy beyond what is achievable with a single model. RF and GB can handle nonlinearity and interactions among predictors automatically through their ensemble structure, where multiple models explore various subsets of features and their combinations. In addition, RF and GB inherently perform feature selection during model training by preferentially splitting on the most informative features, thereby automatically identifying and utilizing the most relevant predictors for the model. Although the results of RF and GB are not directly interpretable regarding predictors due to their ensemble nature, high dimensionality, non-linearity, and varied interactions across trees, interpretable ML methods exist for both RF and GB to answer the following questions (e.g., Apley & Zhu, 2020; Friedman & Popescu, 2008; Molnar, 2019): What are the most important predictors?, to what extent does each predictor on average explain an outcome?, is the relationship between the outcome and a predictor linear, monotonic or more complex?, and which predictors interact?. Park et al. (2023) showed the applicability of RF and GB using person- and item-level predictors to predict person-by-item binary responses. In addition, the authors showed via simulation studies that the model accuracy of RF and GB decreased with increasing levels of random effects over persons. This result highlights the importance of considering random effects in the ensemble approaches. In this study, we focused on RF because Park et al. (2023) showed that the prediction accuracy of RF and GB were similar in the context of predicting person-by-item binary responses.

In the statistics and biomedical informatics literature, methods have been suggested to incorporate RF into the linear mixed model (LMM) or generalized linear mixed model (GLMM). These hybrid methods combine the advantages of RF with the capability to model the correlation due to clusters using LMM or GLMM by replacing the fixed components of LMM or GLMM with RF. As an example of the hybrid model with RF, Hajjem et al. (2014) added RF to LMM for clustered data and demonstrated substantial improvements in prediction accuracy over RF when the random effects to model the correlation are non-negligible in the simulation study. In Ngufor et al. (2019) and Speiser et al. (2019), RF was incorporated into GLMM to account for correlations due to clusters (persons) in the binary longitudinal data. Simulation studies in Ngufor et al. (2019) and Speiser et al. (2019) showed that the hybrid model provided similar or superior prediction accuracy compared to RF and GLMM. These studies highlighted the importance of incorporating random effects to leverage the advantages of RF when its performance is compared with GLMM and RF alone. However, these three prior works did not consider crossed random effects over persons and items in the person-by-item data to develop explanatory models for person-by-item binary responses. In addition, although Ngufor et al. (2019) considered the feature importance measure (Breiman, 2001) to indicate how much each variable contributes to the accuracy of the prediction, multiple interpretable ML methods were not demonstrated in these three prior works. Instead of using random effects to account for unmodeled variability across persons and items, nominal person and item identifiers can be included in the RF to capture this variability. However, including these identifiers substantially increases the feature space, potentially reducing model interpretability particularly when interpreting results related to person- and item-level predictors is of interest.

### 1.3 Study purpose and novel contributions

This study aims to explain outcomes (person-by-item binary responses) by modeling the potential nonlinear and interacted fixed-effects of person- and item-level predictors through the RF in combination with an item response model. The novelty of our approach lies in the incorporation of RF into an item response model as EIRM. This hybrid approach, called EIRM-RF, is expected to model complex nonlinear and interacted fixed-effects of person- and item-level predictors (a main modeling component of RF), while allowing for random effects over persons and items (crossed random effects; a main modeling component of EIRM). It is expected that EIRM-RF will provide improved accuracy to explain variability in item responses compared to EIRM or RF, in the presence of complex nonlinear and interacted effects of the predictors and non-negligible random effects. EIRM-RF differs from the hybrid approach proposed by Pliakos et al. (2019), in which an item response model is first fitted to obtain latent ability scores using existing item response data. Subsequently, a regression tree-based ML method is applied, regressing the obtained ability scores on a set of features characterizing the learners (i.e., person predictors such as demographic information, but excluding item predictors).

In the current study, we focus on using EIRM-RF to explore complex nonlinear and interaction effects among person- and item-level predictors, rather than employing it as a model that incorporates item and person identifiers as features. Interpretable ML derived from RF in EIRM-RF can be used to investigate which person- and item-level predictors are important in explaining item responses, to what extent each predictor on average explains an outcome, the relationship between the outcome and a predictor is linear, monotonic or more complex, and which predictors interact. The estimation of EIRM-RF is implemented using the bimm package (Speiser et al., 2019) in R (R Core Team, 2023), with modifications wherein a standard RF algorithm is incorporated within the framework of Laplace approximation for person-by-item binary responses for crossed random effects (random effects over persons and items).

In addition to model specification and estimation, EIRM-RF is illustrated using a motivating empirical dataset and compared with both EIRM and RF. Furthermore, this study presents the results of simulation studies to investigate the added value of modeling nonlinear and interacted fixed-effects in EIRM-RF compared to EIRM. It also aims to demonstrate the added value of including crossed random effects in EIRM-RF compared to RF alone. In addition, interpretable ML methods are evaluated when the data-generating model is EIRM-tree (i.e., EIRM-RF with a single “true” tree structure).

The rest of this article is organized as follows. In Section 2, EIRM-RF is specified, following presentations of EIRM and RF, respectively. In addition, the estimation method, interpretable ML methods, and evaluation measures for EIRM-RF are presented. In Section 3, EIRM-RF is illustrated using an empirical data set, with comparisons to EIRM and RF. In Section 4, simulation studies are presented to investigate the model accuracy of EIRM-RF and to evaluate interpretable ML methods. The simulation study compares the model accuracy of EIRM-RF, EIRM, and RF under various simulation conditions that affect model performance. In Section 5, we conclude with a summary and a discussion.

## 2 Methods

This section begins by specifying EIRM and RF, respectively, followed by the hybrid model, EIRM-RF. Subsequently, the estimation method, interpretable ML methods, and evaluation measures for EIRM-RF are presented.

### 2.1 Model specifications

#### 2.1.1 Explanatory item response model (EIRM)

The EIRM with linear effects (De Boeck & Wilson, 2004) can be written as 
(1)

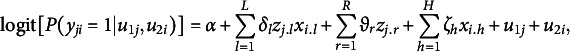

where *j* is an index for person ( 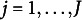 ), *i* is an index for item ( 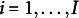 ), *l* is an index for a predictor of person-by-item interaction ( 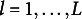 ), *r* is an index for a predictor of person ( 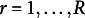 ), *h* is an index for a predictor of item ( 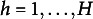 ), 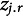 is a person predictor *r*, 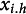 is an item predictor *h*, 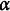 is an intercept (a logit for the probability of a correct response when all predictors are 0), 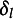 is a fixed effect of a person-by-item predictor *l*, 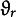 is a fixed effect of a person predictor *r*, 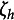 is a fixed effect of an item predictor *h*, 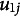 is a random effect over persons, and 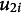 is a random effect over items. The random effects, 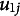 and 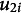 , are considered to account for correlations due to clusters of persons and items, respectively, (De Boeck, 2008). They are assumed to follow a normal (*N*) distribution: 
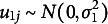
 and 
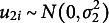
 . In Equation 1, an *linear* relationship is assumed between 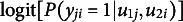 and predictors.

When no predictors are considered in Equation 1, it reduced to a 1-parameter item response model with random item parameters (De Boeck, 2008). The following conditional intraclass correlation (ICC; e.g., Cho & Rabe-Hesketh, 2011) is a useful measure to quantify correlations (Corr) in latent responses due to cross-classified clusters of persons and items, using a 1-parameter item response model with random item parameters. Denote a latent response by 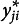 so that the observed response 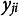 is 1 if 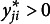 and 0 otherwise. The conditional ICC for persons, correlation among latent responses for the same person conditional on the random effects for items, is calculated as follows: 
(2)

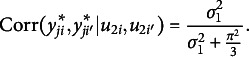

And the conditional ICC for persons, correlation among latent responses for the same item conditional on the random effects for persons, is obtained as follows: 
(3)

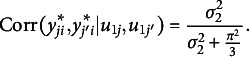



#### 2.1.2 Random forest (RF)

In this study, RF is used for binary data (incorrect response versus correct response as in Park et al. [(Park et al., 2023)]) to explain person-by-item binary item responses. The decision tree algorithm (e.g., Breiman et al., 1984) recursively partitions the feature space into regions by selecting the feature and split point that maximizes the purity or homogeneity of the resulting subsets. Specifically, the decision tree algorithm selects the best feature *k* and split point *s* to optimize the purity or homogeneity of the partitions it creates. The objective at each node *t* is: 
(4)

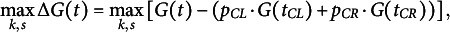

where 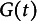 represents the impurity measure of the node before the split, 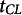 and 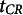 are the child nodes resulting from the split to the left and right, respectively, and 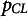 and 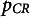 are the proportions of the samples that go to the left and right child nodes, respectively. The partitioning process is repeated until a stopping criterion is met, such as reaching a maximum tree depth or when further splits do not significantly improve purity or homogeneity.

RF proceeds by bootstrapping the data, fitting a decision tree to each bootstrapped dataset, and finally averaging the outputs (i.e., the posterior probability of belonging to an “incorrect” class versus a “correct” class) from multiple decision trees, similar to bootstrap aggregation (bagging; Breiman, 1996). In contrast to bagging, which uses all predictors and leads to similar decision trees, RF reduces correlations among the trees by employing a randomly sampled subset of predictors for each split. This random selection of predictors improves variance reduction.

The motivation for using RF in this study stems from its capability to model nonlinearity and interactions, as well as to automatically select important features, which can be challenging when applying EIRMs. As an ensemble method consisting of decision trees, RF incorporates interactions into the resultant tree structure, wherein the split on one predictor is dependent on the value of another predictor. In addition, each tree in RF produces step functions, which arise from its hierarchical and binary decision-making structure. In this structure, data are split into distinct partitions, with each partition being associated with a specific prediction. Averaging across multiple step functions in RF can lead to a smooth curve between a predictor and outcomes, especially in the presence of nonlinearity (see p. 139 for a detailed illustration in Jacobucci et al., 2023). Furthermore, the random selection of feature subsets for tree splits in RF, combined with the ability to evaluate and prioritize features based on their contribution to the model accuracy, helps in identifying the most significant features. This approach reduces the influence of irrelevant or redundant features and improves the model’s overall predictive performance.

There are two tuning parameters for RF, meaning that there are no analytic formulae to calculate appropriate values. The first tuning parameter is the number of randomly selected predictors at each split time, commonly called 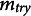 . The second tuning parameter is the number of trees. The optimal value of 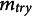 and the number of trees is selected using the manually chosen values. Following a suggestion by Kuhn and Johnson (2018, p. 200), we start with 1,000 trees and increase the number until model accuracy levels off. The grid search systematically evaluates every combination of the candidate values for 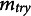 and the number of trees. In addition to 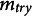 and the number of trees, tree depth can be indirectly controlled by specifying the minimum number of observations required in a terminal node. For classification tasks, the default setting of 1 in software packages (e.g., ranger [Wright & Ziegler, 2017]) is often used, allowing the trees to grow to their maximum depth. This parameter ensures that each tree captures the full complexity of the data unless explicitly restricted. For model tuning, the *k*-fold cross-validation (CV) procedure can be utilized in RF. For small or moderate sample sizes, repeated *k*-fold CV is recommended due to its favorable bias and variance properties, as opposed to simple *k*-fold cross-validation (Kuhn & Johnson, 2018, p. 78). Repeated cross-validation involves performing *k*-fold cross-validation (e.g., 5-fold or 10-fold) multiple times, each with different random splits of the data. By repeating the cross-validation process multiple times, it provides a more stable and reliable estimate of the model’s performance by averaging the results across the repetitions, thereby reducing the impact of variability in any single data split.

#### 2.1.3 Explanatory item response model-random forest (EIRM-RF)

In EIRM-RF, the predicted values from RF using person- and item-level predictors are incorporated into an item response model, while allowing for random effects over persons and items. The EIRM-RF is specified as follows: 
(5)

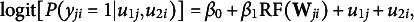

where 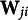 is a matrix of person- and item-level predictors, 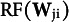 is the predicted values from RF algorithms of each person *j* and item *i* using 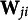 , 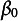 is the (fixed) intercept (a logit for the probability of a correct response when all predictors are 0), and 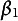 is the effect of the predicted values by RF. Here, one can see that the fixed effects in EIRM, 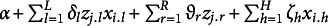 in Equation 1, are replaced with 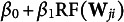 in EIRM-RF, assuming that 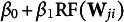 captures the fixed component of EIRM. The cross-classified random intercepts across persons and items, 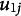 and 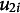 , are considered deviations from the fixed effects of 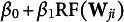 using all predictors, as in a typical GLMM that includes both fixed and random components. In Equation 5, the effects, 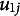 and 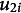 , are assumed to be random and uncorrelated with predictors, as in EIRM or GLMMs, and the (crossed) random effects fully explain intra-cluster (person or item) correlations.

The unexplained variability in the logit-transformed predicted probability based on the results of EIRM-RF can be calculated using the following equation: 
(6)

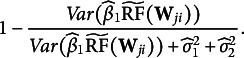



### 2.2 Estimation methods

The algorithm from the bimm package (Speiser et al., 2019), originally developed for binary longitudinal data with a random intercept, was adapted for use in the EIRM-RF with modifications. The modifications include tuning parameters of RF ( 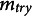 and the number of trees) and a variable importance measure with impurity. A main idea of estimation is to disassociate the fixed-effect component from RF from the random-effect component and iteratively estimate each component within the framework of Laplace approximation for crossed random effects (random effects over persons and items). Cho et al. (2012) demonstrated that Laplace approximation recovers the variances of random effects well in the 1-parameter item response model with random item parameters, provided the number of clusters (i.e., the number of persons and items) is greater than 10.

In implementation, recursive partitioning for RF was used to estimate the fixed-effects of EIRM-RF assuming that the random effects are known, and then estimate the random effects assuming the fixed-effects estimated by the recursive partitioning in the previous iteration is correct. Specially, the algorithm, as implemented in the bimm package, involves two main steps:

Step 1. Initialization of RF and EIRM-RF.

- Step 1.1: Fit RF using 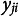 and 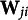 , and calculate the predicted 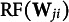 . The ranger function in the ranger package in R was used to fit RF. The split criterion used is the Gini-based impurity.
- Step 1.2: Fit EIRM-RF (Equation 5) to obtain the random effects, 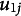 and 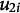 . The glmer function in the lme4 package (Bates et al., 2015) in R was used to fit EIRM-RF.
- Step 1.3: Calculate the predicted probability using EIRM-RF (Equation 5) based on the predicted 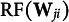 and the predicted random effects for person *j* and item *i*, denoted by 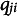 .

Step 2. Iterations through the following steps until convergence.

- Step 2.1: Calculate the target outcome 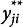 and generate a binary response based on the following equation with a split function 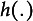 (defined below) and the predicted probability 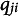 from Step 1: 
(7)

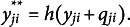

The value of 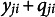 ranges from 0 to 2 because 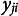 is either 0 or 1 and 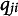 being a probability, ranges from 0 to 1. from 0 to 1. The following split function is used to dichotomize the predicted probabilities (Speiser et al., 2019): 

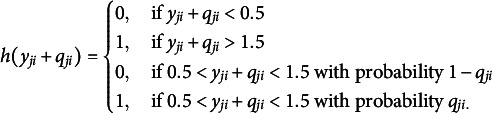

In this split function, if the combined value is between 0.5 and 1.5, the outcome is determined probabilistically, depending on 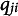 . When the original binary response 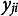 aligns with the predicted probability from the iteration, the target outcome 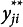 is set to the original binary response 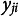 .
- Step 2.2: Repeat Step 1 using 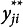 as the outcome until the out-of-bag [OOB] prediction error stabilizes within the fourth decimal point across iterations.

### 2.3 Interpretable ML

Based on results of RF in EIRM-RF, the following four interpretable ML methods were considered. The first method is a feature importance measure (Breiman, 2001). In this study, Gini-based importance was calculated during the construction of the RF. Gini-based importance measure is a metric used to quantify the significance of each predictor in the classification process of RF (incorrect response versus correct response). It measures the prediction strength of each predictor. Specifically, the Gini impurity is calculated for each predictor across all trees by summing the decrease in Gini impurity achieved by each split that involves that predictor. Predictors with higher Gini importance scores are considered more important in the classification process, as they contribute more to reducing impurity and separating the classes effectively. The feature importance measure and plot were obtained using the vip function of the vip package (Greenwell & Boehmke, 2020).

The second method is a partial dependence plot (Breiman, 2001), where the *x*-axis represents the range of values for a specific predictor, and the *y*-axis indicates the average predicted outcome in a ML method (RF in the current study) after accounting for the effects of other predictors, assuming they are independent. This implies that the nature of the relationship (e.g., linear, monotonic, or more complex) between the continuous predictor and the predicted outcome can be biased in the presence of correlations among predictors. However, the partial dependence plot provides a global overview of the effect of a predictor across its entire range, regardless of the distribution of other predictors. Therefore, in this study, the partial dependence plot was used to understand how each predictor relates to the predicted outcome. The partial dependence plot was obtained using the FeatureEffect function of the iml package (Molnar et al., 2018).

As the third method, an accumulated local effect plot (Apley & Zhu, 2020) addresses the limitations of the partial dependence plot by focusing on how the predictions change locally as a feature varies. The accumulated local effect plot computes the differences in predictions over small intervals of the feature of interest, thereby capturing the local effect of this feature. Thus, the accumulated local effect plot can more effectively handle correlated features in the presence of interactions and correlations among predictors than the partial dependence plot can. In this study, accumulated local effect plots were considered to identify the nature of the relationship (e.g., linear, monotonic, or a more complex pattern) between the continuous predictor and the predicted outcome. Although the accumulated local effect plot does not explicitly indicate specific cut points or ranges on the *x*-axis that could optimize the model’s purity in the context of RF, this plot is primarily designed to provide a general understanding of the relationship between a predictor and the outcome by averaging over all trees in the forest, rather than focusing on the specific decision boundaries or splitting points that individual trees may use. However, the accumulated local effect plot can highlight regions where the relationship between the predictor and the outcome is particularly strong or weak, which might suggest areas where splitting could improve model performance. In RF, the actual splitting points are determined by maximizing the reduction in impurity (Gini impurity in the current study), and these points may vary across different trees due to the random nature of the algorithm. The accumulated local effect plot was created using the FeatureEffect function of the iml package.

The fourth method is *H*-statistic (Friedman & Popescu, 2008) to quantify to what extent the prediction is the result of joint effects of the predictors. The *H*-statistic considers how much of the variance in model predictions can be attributed to the interactions between the features in question, relative to the total variance in predictions. As a result, the *H*-statistic ranges from 0 to 1. A value of 0 indicates no interaction between the predictors, while a value of 1 suggests a strong interaction. The *H*-statistic was obtained using the Interaction function of the iml package.

### 2.4 Evaluation measures

For model accuracy, two evaluation measures, the area under the receiver operating characteristic curve (AUC) and the Brier score (OOB prediction error), were considered. The AUC was calculated by plotting the true positive rate against the false positive rate at various threshold settings and then computing the area under this curve. For predictions that are completely random, the AUC value is typically around 0.5, and a value of 1 indicates perfect predictions by a model. In addition, the Brier score was calculated as 
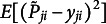
 , where 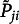 is the predicted probability for person *j* and item *i* by EIRM, RF, or EIRM-RF. The Brier score (OOB prediction error) is computed by passing the unsampled observations through the trees and the final Brier score is obtained by averaging the results from each tree. The smaller the Brier score, the better the model accuracy.

## 3 Illustration

In this section, EIRM-RF is illustrated with the comparison of EIRM and RF.

The primary research questions in applying EIRM-RF, EIRM, and RF are: (a) which person- and item-level predictors are important in explaining person-by-item binary item responses?, (b) to what extent does each predictor, on average, explain an outcome?, and (c) is the relationship between the outcome and a predictor linear, monotonic, or more complex?, and (d) which predictors interact?

### 3.1 Data description

The illustrative data set is from Goodwin et al. (2020) in which EIRM was applied using the person-level predictors. The dataset from Goodwin et al. (2020) was chosen because it contains a relatively large number of person-level and item-level predictors compared to other test data sets. Goodwin et al. (2020) did not consider the method developed in the current study.

#### 3.1.1 Study design and outcome variable

Goodwin et al. (2020) implemented a study design incorporating both within- and between-subjects factors to explore how students approach reading in paper and digital mediums. Each student served as their control in this design, being randomly allocated to one of two conditions that determined the medium (paper or digital) for reading the first (part 1) or second part (part 2) of the passage. In condition A, students read the first part on paper and the second part digitally, while in condition B, the order was reversed. This approach enabled an investigation into the impact of the reading medium on the outcomes, while controlling for potential practice effects related to the text.

In Goodwin et al. (2020), the study assessed reading comprehension using 14 questions, excluding one general question (Item 1) linked to both part 1 and part 2. Where feasible, questions from the National Assessment of Educational Progress (NAEP) were employed, with five of seven NAEP questions included. All 14 questions were scored as an incorrect response (0) or a correct response (1). All 381-participants, regardless of their experimental condition, received the same 14-item test. As a result, outcome variables are the person-by-item binary item responses.

Below, the person-level and item-level predictors are presented. Table 1 shows the summary and descriptive statistics of 14 person-level predictors and three item-level predictors.

**Table 1:** Empirical study: descriptive statistics of predictors.

Predictors	Mean (SD)	Frequency (%)
**Person level**		
*Backgrounds*		
Grade		
- Grades 5–7		34.65
- Grade 8		65.35
Preference		
- Paper		24.41
- Digital		36.48
- Both		38.58
- Missing		0.52
Condition		
- Condition A		50.13
- Condition B		49.87
Ethnicity		
- Non-White		56.69
- White		43.31
Pretest total scores ( 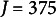 )	3.291 (1.329)	
*Reading behaviors*		
No. of digital highlighting ( 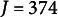 )	2.158 (4.495)	
No. of paper highlighting ( 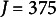 )	4.461 (8.612)	
Looking back digitally		
- No		8.92
- Yes		90.29
- Missing		0.79
Looking back on paper		
- No		13.12
- Yes		86.09
- Missing		0.79
Use of dictionary in digital		
- No		74.28
- Yes		25.72
No. of annotating digitally ( 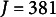 )	0.073 (0.429)	
No. of annotating on paper ( 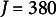 )	0.408 (1.427)	
No. of mouse action ( 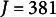 )	22.593 (28.379)	
Total response time in mins ( 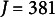 )	4.598 (2.140)	
**Item level**		
Task		
- locate and recall		57.14
- critique and evaluate		7.14
- integrate and interpret		35.71
Format		
- multiple choice		42.86
- open response		35.71
- true false		21.43
Part		
- Part 1		50
- Part 2		50

*Note:* The response time in minutes typically follows a skewed distribution. However, in the current study, the median total response time for solving 14 items is 4.15 minutes. This suggests that the skewness is not severe.

#### 3.1.2 Person-level predictors

In addition to the study design condition (condition A versus condition B; conditionb), there are four background, person-level predictors: grade level (Grade 8 versus Grades 5–7; grade8), preference (paper, digital, or both; preference), ethnicity (white non-white; white), and pretest total scores (from 10 items; pretotal) to measure pre-knowledge of the contents. Furthermore, there are seven person-level reading behavior predictors in Goodwin et al. (2020): digital and paper highlighting (ndighighlight and npaphighlight), and digital and paper annotating (nanndigital and nannpaper) which were coded for the number of occurrences, content, and link to the part (section of the text) and areas of interest. In addition, the use of a digital dictionary (dictionarydig) and whether students looked back at the digital and/or paper text while answering questions on the posttest were coded for occurrence (did not occur versus occurred; lbackdigital and lbackpaper). Beyond these seven reading behavior person-level predictors, this study considered mouse actions which were coded for the number of occurrences while reading parts 1 and 2 (mouseaction). Furthermore, the total response time (in mins) to solve 14 items (responsetime) was also considered.

#### 3.1.3 Item-level predictors

In addition to the passage (part 1 part 2; part) from the study design, the 14-items have two more item attributes, item format (format) and item task (task). Of the 14-items, there are six multiple-choice items, five open-ended items, and three true/false items. The test, balanced according to the NAEP framework, evaluated reading comprehension through eight tasks of “locate and recall”, five tasks of “integrate and interpret”, and one task of “critique and evaluate”.

### 3.2 Data preprocessing

For EIRM, RF, and EIRM-RF, data were arranged in the long format, with each row representing a unique pair of a person and an item. Missing observations (constituting 12.7% of the data) were excluded from the analyses in the long format, which leads to 361 persons and 14 items. The continuous person-level predictors were centered at the means. The categorical predictors were coded as dummy variables in EIRM. However, they were considered nominal variables in RF and EIRM-RF because the RF algorithm makes splits based solely on category membership (Jacobucci et al., 2023).

There was one near zero-variance predictor (the number of annotating in digital; nanndigital), which may cause issues when subsampling in RF. Thus, the predictor was not considered in EIRM, RF, and EIRM-RF. In addition, there were no highly correlated predictors ( 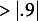 ), nor were there any linear dependencies between predictors. The median of the correlations among predictors is 0.003, and the interquartile range is 0.070.

### 3.3 Analysis

The ICCs based on the EIRM without any predictors (i.e., 1-parameter item response model with random item parameters) were 0.173 and 0.166 for persons and items, respectively. These ICCs indicate that there are non-negligible correlations in latent responses due to cross-classified clusters of persons and items.

For comparison purposes, EIRM, RF, and EIRM-RF were fit to the same empirical data set. For the RF approach, we fit both RF with person- and item-level predictors and one with person- and item-level identifiers. In fitting EIRM, linear and no-interaction effects of person- and item-level predictors were considered, which is a common practice in applications (see Baayen et al., 2008; De Boeck & Wilson, 2004; Steacy, 2020). The comparison of EIRM with EIRM-RF underscores the addition of potential nonlinearity and interaction effects in EIRM-RF. In addition, the comparison between RF with person- and item-level predictors and EIRM-RF highlights the modeling of random effects over persons and items. The RF model with person- and item-level identifiers was used to compare its accuracy with that of EIRM-RF. The glmer function from the lme4 package and the ranger function from the ranger package were used to fit EIRM and RF, respectively. The estimation method, as outlined in Section 2, was employed to fit the EIRM-RF model. In building RF and RF in EIRM-RF, the splitting rule was based on Gini impurity. In addition, the minimal node size was set to 1, which means that the trees are grown until all leaves either contain only one observation or are completely homogeneous.

For model tuning of RF with person- and item-level predictors and one with person- and item-level identifiers, a five repeated 10-fold cross-validation approach was implemented. The model tuning with selected values was conducted to optimize the two tuning parameters ( 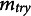 and the number of trees) using the caret package (Kuhn, 2008). The four values of 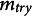 (2, 3, 4 [the square root of the number of predictors as the recommended value for 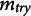 ], and 5) and the five values of the number of trees (1000, 1500, 2000, 2500, and 3000) were selected. In RF, it is common practice to explore a range of 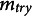 values. Smaller values of 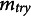 can lead to more diverse trees, which generally improve the model’s robustness. The values selected (2, 3, 4, or 5) for both item- and person-level predictors represent a reasonable range to explore based on this principle, ensuring the model is neither too simple nor too complex (Breiman, 2001). The optimal model, defined as the one with the highest accuracy, was selected with 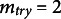 and the number of trees = 2500 for RF with person- and item-level predictors; and with 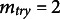 and the number of trees = 1000 for RF with person- and item-level identifiers. RF was rebuilt using these optimal values for the final model. For EIRM-RF, AUC, and Brier scores differs in the second decimal point with the varying values of 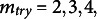 and 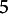 and the number of trees 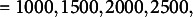 and 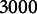 . Thus, 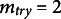 and the number of trees=1000 were used in RF of EIRM-RF. The EIRM-RF converged at the 19th iteration.

### 3.4 Results

Table 2 presents the AUC and Brier scores for EIRM, RF, and EIRM-RF. EIRM, with an AUC of 0.800 and a Brier Score of 0.179, provided a baseline that lacks the nonlinear and interaction effects of the predictors. In contrast, EIRM-RF, which incorporates both nonlinearity and interactions, showed improved performance, evidenced by a higher AUC of 0.910 and a lower Brier Score of 0.113. RF with person- and item-level predictors demonstrated performance with an AUC of 0.880 and a Brier Score of 0.167, which were lower and higher, respectively, compared to the AUC of 0.910 and Brier Score of 0.113 for EIRM-RF. This improvement suggests that the ability of EIRM-RF to effectively model random effects contributes to its enhanced predictive accuracy. Based on the results of EIRM-RF, the unexplained variability in the logit-transformed predicted probability using Equation 6 was 0.571. The accuracy of RF with person- and item-level identifiers was higher than that of both EIRM and RF with person- and item-level predictors. However, it is slightly lower than that of EIRM-RF.

**Table 2:** Empirical study: accuracy of EIRM, RF, and EIRM-RF

Models	Modeling components		Evaluation measures	
	Nonlinearity & interactions	Random effects	AUC	Brier score
EIRM		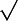	0.800	0.179
RF with predictors	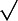		0.880	0.167
RF with identifiers	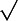		0.899	0.132
EIRM-RF	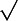	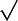	0.910	0.113

For comparison with EIRM-RF, Table 3 shows the results of EIRM. Among background person-level predictors, grade level, being white, and the pretest total scores were statistically significant ( 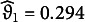 , 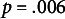 for grade; 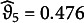 , 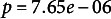 for white; 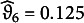 , 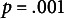 for pretest total scores). Being in the eighth grade (as opposed to grades 5–7), being white, and having a higher pretest total score led to a higher probability of a correct response. Among reading behavior person-level predictors, looking back on paper and the response time (for all items) were statistically significant ( 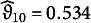 , 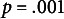 for looking back on the paper; 
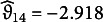
 , 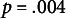 for the response time). It was found that looking back on the paper and spending less time solving all items were associated with a higher probability of a correct response. None of the effects for the item-level predictors were significant in EIRM.

**Table 3:** Empirical study: results of the EIRM

	EST	SE	*z*	*p*-value
**Fixed effects**				
intercept[ 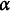 ]	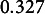	0.730	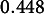	.654
grade8[ 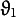 ]	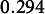	0.107	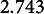	.006
preference0[ 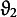 ]	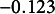	0.134	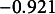	.357
preference1[ 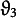 ]	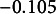	0.114	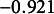	.357
conditionb[ 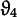 ]	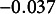	0.115	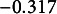	.751
white[ 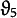 ]	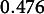	0.106	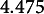	7.65e-06
pretotal[ 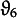 ]	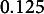	0.038	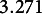	.001
ndighighlight[ 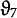 ]	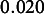	0.015	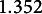	.176
npaphighlight[ 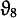 ]	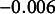	0.006	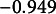	.343
lbackdigital[ 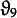 ]	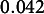	0.183	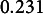	.817
lbackpaper[ 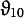 ]	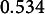	0.155	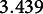	.001
dictionarydig[ 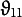 ]	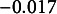	0.122	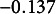	.891
nannpaper[ 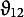 ]	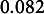	0.043	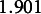	.057
mouseaction[ 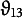 ]	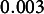	0.002	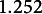	.211
responsetime[ 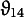 ]	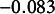	0.029	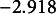	.004
task2[ 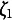 ]	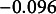	0.834	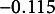	.909
task3[ 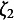 ]	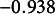	0.796	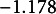	.239
format2[ 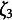 ]	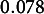	0.477	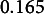	.869
format3[ 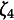 ]	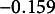	0.530	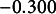	.764
part[ 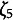 ]	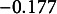	0.404	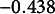	.661
**Random effects**				
Var of 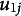 [ 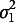 ]	0.486			
Var of 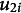 [ 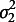 ]	0.473			

*Note:* The grade 8 is the dummy predictor for Grade 8, with Grades 5–7 as the reference level; The preference0 is the dummy predictor for paper preference, with a preference for both paper and digital as the reference level. The preference1 is the dummy predictor for digital preference, again with a preference for both paper and digital as the reference level. The conditionb is the dummy predictor for Condition B, with Condition A as the reference level; The white predictor is the dummy predictor for being white, with not being white as the reference level. The task item-level predictor has three categories: the task2 represents the dummy predictor for the “locate and recall” task, with “critique and evaluate” as the reference level; similarly, task3 indicates the dummy predictor for the “integrate and interpret” task, also with “critique and evaluate” as the reference level; The format item-level predictor also has three categories: format2 represents the dummy predictor for the “open response” format, using the “multiple choice” format as the reference level, and format3 represents the dummy predictor for the “true/false” format, also using the “multiple choice” format as the reference level; the part item-level predictor is the dummy predictor for part 2, with a reference of part 1.

**Figure 1:**
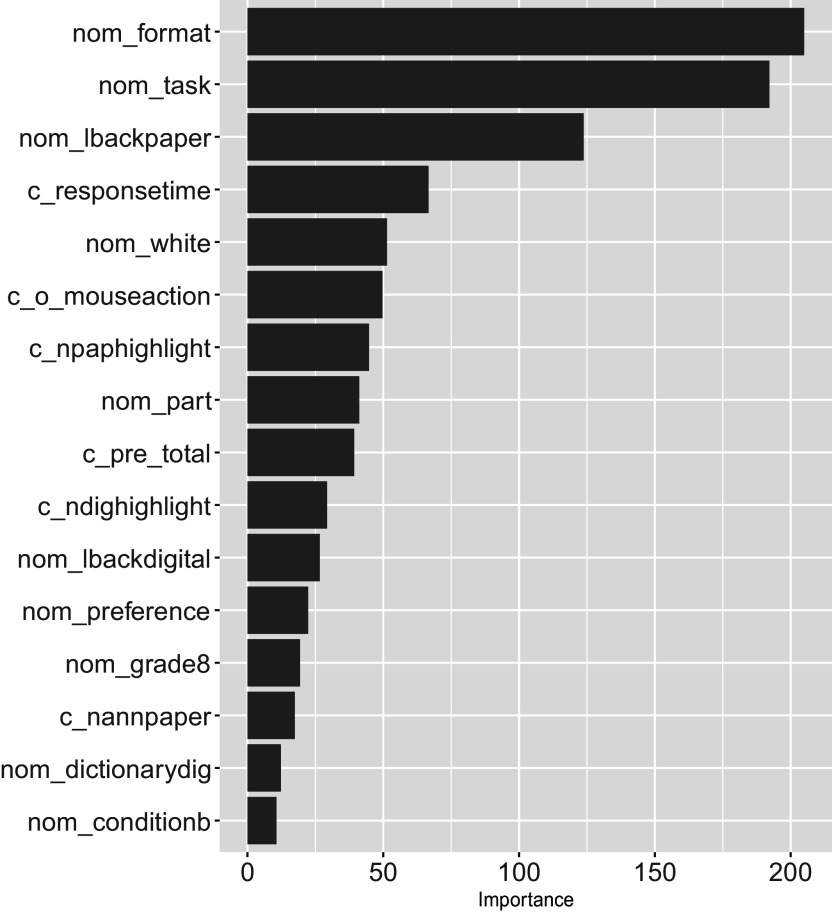
Empirical study: feature importance measures for EIRM-RF. *Note*: In the predictor (feature) name, “nom” indicates a nominal predictor, and “c” indicates the mean-centered predictor.

Because the EIRM-RF exhibited the highest accuracy among EIRM, RF, and EIRM-RF, its results were further interpreted using interpretable ML methods. Figure 1 displays the plots of feature importance measures for EIRM-RF. As shown in Figure 1, item format and task emerged as the two most influential predictors, suggesting that the format of items and the nature of tasks significantly improve the model’s predictions. The three reading behavior predictors – looking back on paper, total response time (for all items), and mouse action – followed item format and task were the relevant predictors. Among the background predictors, ethnicity (white non-white) was the most important.

**Figure 2:**
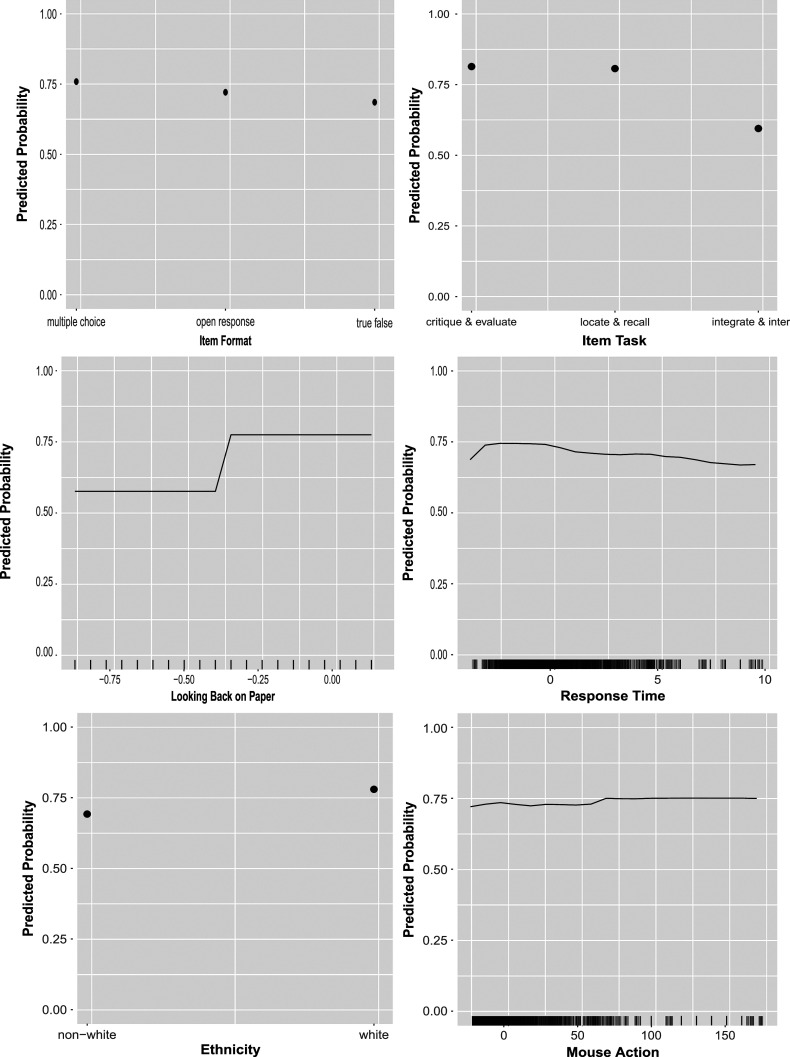
Empirical study: partial dependence plots from EIRM-RF. *Note*: Continuous predictors were mean-centered; each tick mark in the *x*-axis represents values of a continuous predictor.

**Figure 3:**
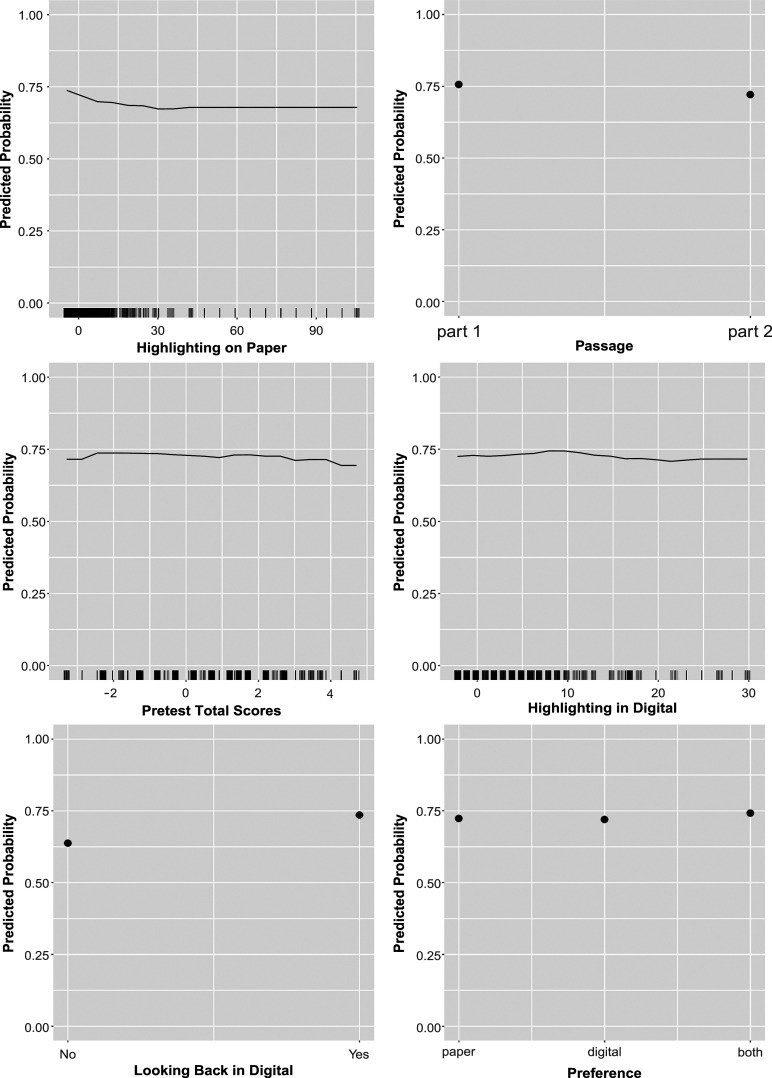
Empirical study: partial dependence plots from EIRM-RF. *Note*: Continuous predictors were mean-centered; each tick mark in the *x*-axis represents values of a continuous predictor.

**Figure 4:**
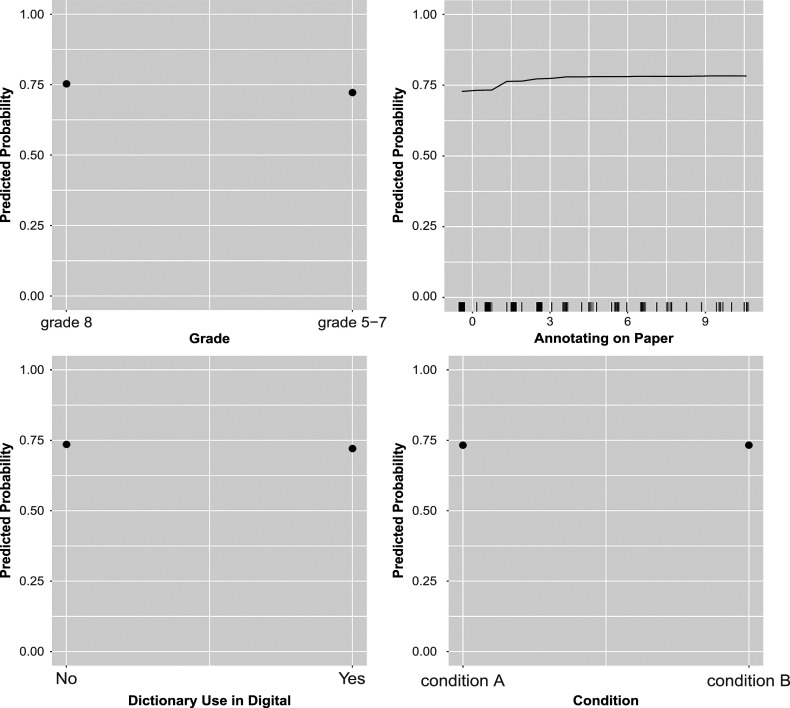
Empirical study: partial dependence plots of EIRM-RF. *Note*: Continuous predictors were mean-centered; each tick mark in the *x*-axis represents values of a continuous predictor.

Figures 2–4 display the partial dependence plots of the predictors in EIRM-RF, which were used to explore the effect of each predictor on the predicted outcome (probability) of a correct response on average, after accounting for the average effects of all other predictors. Regarding item format, the predicted probability was higher for multiple-choice items, followed by open-response and true-false items. For item tasks, the predicted probability was higher for “critique & evaluate” items, followed by “locate & recall” and “integrate & interpret” items. In addition, it was higher in part 1 than part 2. Regarding reading behaviors, the following patterns were observed. The predicted probability increased with looking back both on paper and digitally. The predicted probability decreased as the number of highlights on paper increased, whereas it increased with more highlighting done digitally initially and decreased after reaching 9 highlights. Additionally, the predicted probability initially rose with the total response time (for all items) but decreased after reaching 0, aligning with prior findings on response time (e.g., Bolsinova & Molenaar, 2018). Moreover, an increase in predicted probability was associated with more frequent mouse use and non-dictionary use in digital. There was also an increase in predicted probability with more annotations made on paper. For background predictors, a higher predicted probability was observed for students who were white, in grade 8 (as compared to grades 5–7), and preferred both paper and digital (followed by those who prefer paper and digital, respectively). However, there were no differences in the predicted probability between condition A and condition B. In addition, the predicted probability initially increased with the pretest total scores but decreased after reaching about 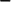 2 of the pretest total scores. These findings align with the larger reading literature (e.g., Coiro, 2021) and show nuances in how these features contribute to reading comprehension in different mediums. For example, whereas paper highlighting does not seem to support comprehension at all, digital highlighting supports comprehension up to a point likely because small amounts of highlights draw attention to important ideas but too many highlights hide those key ideas amongst others.

**Figure 5:**
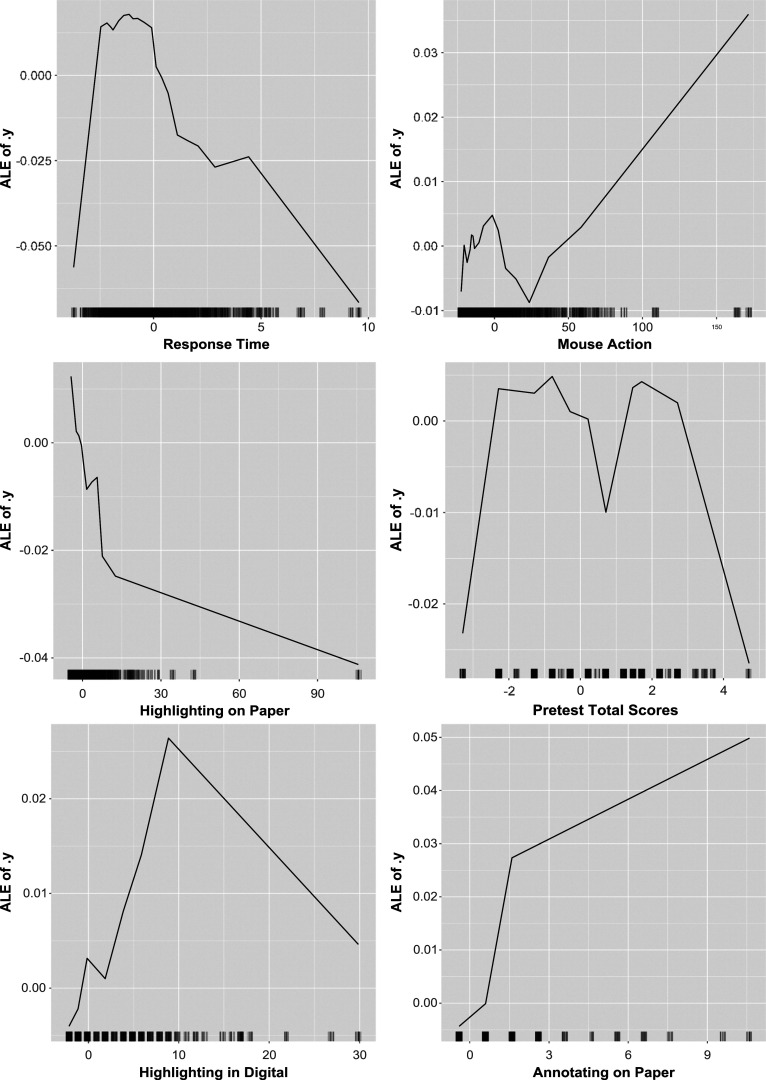
Empirical study: accumulated local plots of EIRM-RF. *Note*: Continuous predictors were mean-centered; each tick mark in the *x*-axis represents values of a continuous predictor.

Figure 5 displays the accumulated local effect plots for the continuous predictors in EIRM-RF. The *y*-axis in these plots represents the accumulated local effect (labeled as ALE on the *y*-axis) on the predicted probability. As shown in Figure 5, the relationships between each predictor and ALE are nonlinear. For example, there is an increasing pattern at lower levels of pre-test scores and a decreasing pattern at higher levels of pre-test scores. Note that the significant decrease in ALE observed for pre-test total scores of 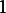 can be attributed to the absence of any scores of 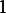 . The effect of pre-test knowledge increases initially because a student with more background knowledge is likely better equipped to build an accurate model of the text, as they are more likely to identify and highlight areas of interest that are relevant to the text in comparison to irrelevant details. However, those who are experts in the topic may already possess a model of the topic that does not align with the model being portrayed in the text. In such cases, the pre-existing knowledge may compete with the new information being learned, thus becoming less supportive of comprehension. These complex and nonlinear relationships presented in Figure 5 might be overlooked when fitting EIRM with linear effects. Therefore, accumulated local effect plots, such as those in Figure 5, can be used to identify the failure to model non-linear effects in EIRM when using only linear effects.

**Figure 6:**
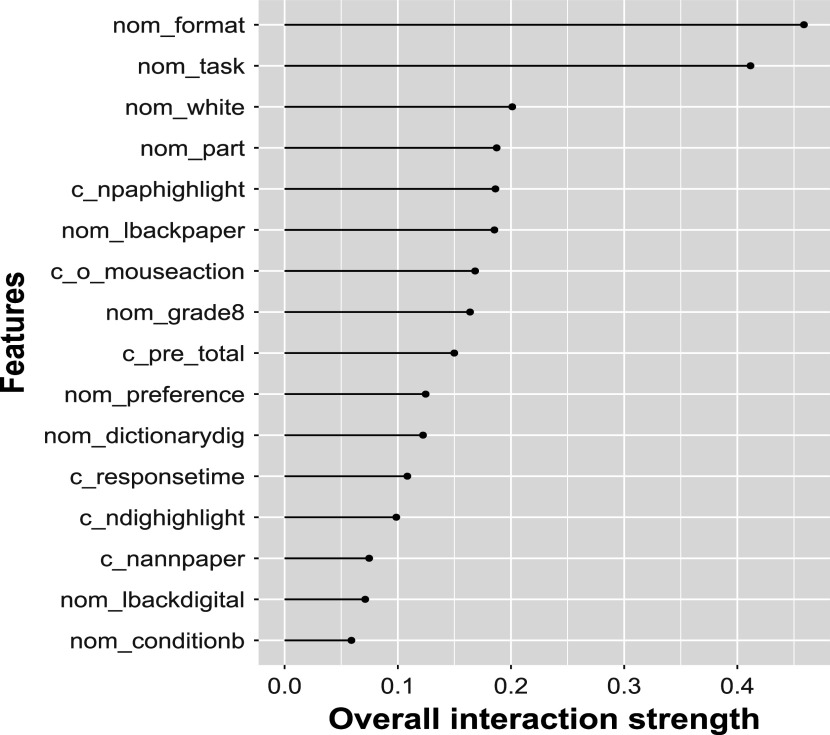
Empirical study: the *H*-statistic of all predictors from EIRM-RF. *Note*: In the predictor (feature) name, “nom” indicates a nominal predictor, and “c” indicates the mean-centered predictor; Values on the x-axis indicate the strengths of the interaction effects.

**Figure 7:**
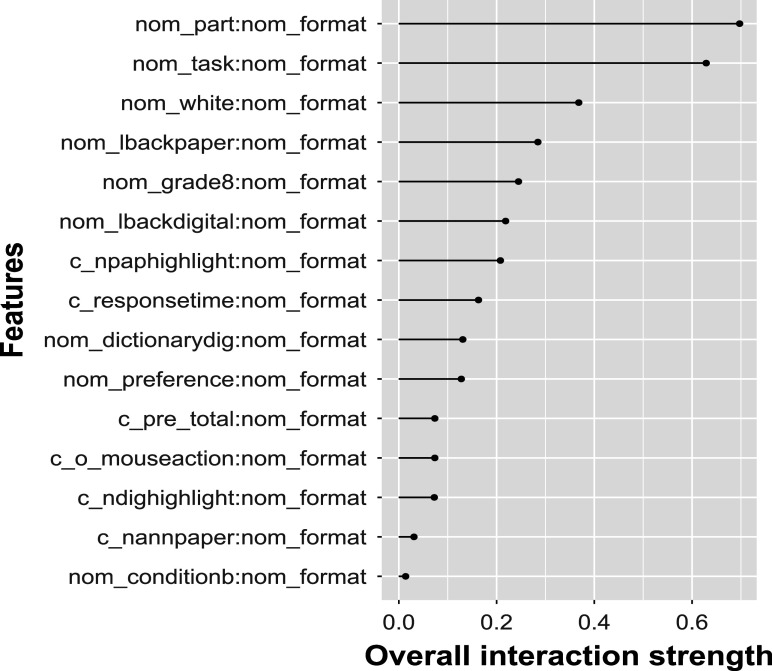
Empirical study: the *H*-statistic of the two-way interactions between the item format and all other predictors from EIRM-RF. *Note*: In the predictor (feature) name, “nom” indicates a nominal predictor, and “c” indicates the mean-centered predictor; Values on the *x*-axis indicate the strengths of the interaction effects.

Figure 6 presents the *H*-statistic for EIRM-RF, quantifying the extent to which the prediction results from the joint effects of the predictors. The item format showed the strongest interaction effect with a value of 0.46, followed by the item task, ethnicity (non white white), and the part (passage). Among the reading behavior person-level predictors, the number of highlighting on paper and looking back on paper exhibited the strongest interaction effects with other predictors. Given that the item format exhibited the strongest interaction signal, the subsequent question that arose was which other variable demonstrated the greatest strength in this interaction. Figure 7 shows the *H*-statistic for the two-way interactions between the item format and all other predictors. The strongest interaction was found to be 0.70 between the item format and the part, followed by interactions between the item format and the item task, ethnicity, and the tendency to look back at the paper.

## 4 Simulation study

In this section, simulation studies are presented to (a) evaluate the model accuracy of EIRM-RF in explaining item responses, compared to that of EIRM and RF with person- and item-level predictors when the data-generating models are EIRMs, and (b) evaluate interpretable ML methods under EIRM-RF and RF when the data-generating model is EIRM-tree (i.e., EIRM-RF with a single “true” tree structure).

### 4.1 Simulation study 1

#### 4.1.1 Simulation designs and expected results

The varying simulation factors of fixed-effect structures and variances of random effects were considered to evaluate the relative performance of EIRM-RF versus EIRM (with linear and no-interactions), and EIRM-RF versus RF with person- and item-level predictors. In addition to these simulation factors, the number of persons and items (sample size) was selected to investigate how the three models behave in small and medium sample sizes. Person-by-item binary responses were generated under EIRM (Equation 1), with the following levels of the three varying simulation factors:

- Fixed-effect structures: simple (linear and no-interactions) and complex (nonlinear and interactions)
  The numbers of person- and item-level predictors were set to be 13 and 3, respectively, as used in the empirical study. In practice, it is common to have a greater number of person-level predictors than item-level predictors (De Boeck & Wilson, 2004). Three item-level categorical predictors ( 
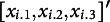
 ) and four person-level categorial predictors ( 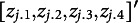 ) were generated with random sampling of 2 ( 
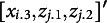
 ) or 3 ( 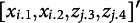 ) categories with replacement. In generating the fixed-effect structures, these categorical predictors were dummy-coded, and the dummy-coded predictors are noted in the third subscript (e.g., the third subscript in 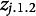 indicates the dummy-coded predictor for the category). In addition, each of the nine person-level continuous predictors 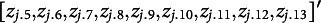 were generated with a standard normal distribution. The same generated predictors were used in both simple and complex fixed-effect structures. Intercept parameter ( 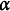 ) was set to be 0. All regression coefficients (on the logit scale) were randomly sampled from a uniform distribution between 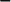 1 and 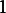 . These ranges were referred to from the EIRM applications when predictors are standardized (e.g., De Boeck & Wilson, 2004). For the simple fixed-effect structure, the linear and main effects of all item-level and person-level predictors were considered in the fixed part of EIRM ( 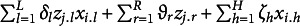 in Equation 1), specified as follows: 

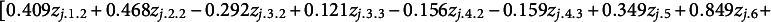




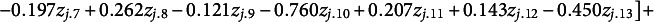



(8)

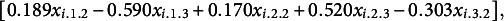

where the terms of the first bracket represent the main effects of person-level predictors, and and the terms of the second bracket represent the main effects of item-level predictors.
  For the complex fixed-effect structure, the following was used in the fixed part of EIRM: 

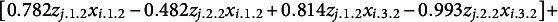




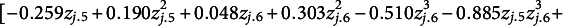




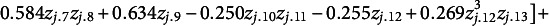



(9)

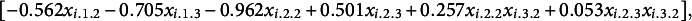

where the terms of the first bracket represent the interaction effects between person-level and item-level predictors, the terms of the second bracket represent the main effects of person-level predictors, and the terms of the third bracket represent the main effects of item-level predictors.
- Variances of random effects: no, small, and large^3^
  For the no-variance level, values of 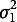 and 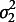 were both set to 0. Although this level may be rare in practice, it was considered to demonstrate the maximal performance of RF and the potential issue with boundary solutions (0 variances) in random effects for EIRM and EIRM-RF. For the small variance level, values of 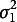 and 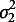 were both set to 0.7. This setting leads to the ICC of 0.176 ( 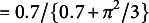 ) for persons and items, respectively. For the large variance level, 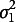 and 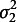 were both set to 2.8, resulting in an ICC of 0.460 ( 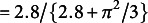 ) for persons and items, respectively.
- The number of persons and items: small and medium
  For the small sample size, a dataset comprising 100 persons and 10 items was considered as used in Park et al. (2023). For the medium sample size, a dataset with 400 persons and 30 items was selected.

The mean and variance of Equation 8 were 0.033 and 2.022, respectively, for the small sample size, and they were 0.387 and 2.030, respectively, for the medium sample size. In addition, the mean and variance of Equation 9 were 0.609 and 2.552, respectively, for the small sample size, and they were 0.538 and 2.952, respectively, for the medium sample size. For both small and medium sample sizes, the unexplained variances in the logit-transformed predicted probability were 0.4 and 0.7 for the small and large variance levels of the simple and complex fixed-effect structures, respectively.

The total number of simulation conditions is 12, comprising levels of fixed-effect structures, three levels of variances of random effects, and two levels of the number of persons and items. For each simulation condition, 1,000 replications were conducted. The fixed effects and the generated predictors were set to the same across the replications, and the random effects were generated at each replication. The EIRM (with the fixed-effects specified in Equations 8 and 9), RF, and EIRM-RF models were fitted to the same generated data sets. Model accuracy was evaluated using both the AUC and the Brier score, consistent with the methods employed in the empirical study.

The model accuracy of EIRM-RF is expected to be better to that of EIRM when a complex fixed-effect structure is modeled through RF in EIRM-RF. In the presence of variances, the model accuracy of EIRM, RF, and EIRM-RF is expected to be better in the simple fixed-effect structure than in the complex fixed-effect structure. When variances of random effects are large, the model accuracy of EIRM-RF, which accounts for such variances, is expected to surpass that of RF. However, RF is expected to outperform both EIRM-RF and EIRM in scenarios with zero-variance in which boundary solutions occur in fitting EIRM and EIRM in EIRM-RF. In addition, across all models — EIRM, RF, and EIRM-RF — model accuracy is anticipated to be better at the large sample size level than at the small sample size level.

#### 4.1.2 Analysis

The generated predictors exhibited no near zero-variances, no highly correlated predictors, and no linear dependencies. In fitting the EIRM, all person-level and item-level categorical predictors were dummy coded, using the first category as the reference, and the dummy-coded predictors representing the other categories were included. In the fitting of RF and EIRM-RF, the person-level and item-level categorical predictors were treated as nominal predictors.

A 5-repeated 10-fold cross-validation for RF model tuning was conducted using 10% of replications in each simulation condition. As in the empirical study, 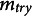 values of 2, 3, 4, and 5, along with 1000, 1500, 2000, 2500, and 3000 trees, were selected for model tuning. In all simulation conditions, the optimal model was selected with 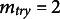 . However, the number of trees ranged from 1,000 to 2,500 across the simulation conditions. For the EIRM-RF, using 10% of replications in each simulation condition, the AUC and Brier scores showed no differences at the second decimal point for the selected 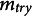 and the number of trees. Therefore, 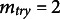 and 1,000 trees were used in all simulation conditions for fitting EIRM-RF in all simulation conditions. In all simulation conditions, convergence was reached in fewer than 30 iterations when fitting EIRM-RF.

#### 4.1.3 Results

The means of the AUC and Brier score across 1,000 replications are presented in Table 4. The following expected patterns were evident in Table 4. First, with variances present, the AUCs were higher and Brier scores were lower for all EIRM, RF, and EIRM-RF in the simple fixed-effect structure compared to the complex fixed-effect structure. Second, while the differences were smaller, the AUCs and Brier scores for EIRM-RF were slightly higher and lower, respectively, compared to EIRM and RF when there was small variance and a simple fixed-effect structure. However, with large variance and a complex fixed-effect structure, EIRM-RF significantly outperformed both EIRM and RF. Third, in the presence of variances, the AUC for EIRM-RF was higher than those of EIRM and RF, and the Brier score for EIRM-RF was lower than those for EIRM and RF. Fourth, at the level of no-variances, the AUC for RF was higher compared to those of EIRM and EIRM-RF, and the Brier score for RF was lower than those for EIRM and EIRM-RF. At the level of no-variances, the AUCs of EIRM and EIRM-RF were close to 0.5, indicating that the two models made predictions randomly. Fifth, the AUC was higher and the Brier score was lower with a medium sample size compared to a small sample size when there were variances.

**Table 4:** Simulation study 1: model accuracy of EIRM, RF, and EIRM-RF

Conditions			AUC			Brier score		
Sample size	Fixed effect	Variance	EIRM	RF	EIRM-RF	EIRM	RF	EIRM-RF
Small	Simple	No	0.535	***0.902***	0.598	0.128	***0.097***	0.125
Small	Simple	Small	0.905	0.903	***0.942***	0.096	0.120	***0.061***
Small	Simple	Large	0.900	0.888	***0.981***	0.110	0.113	***0.017***
Small	Complex	No	0.542	***0.909***	0.501	0.202	***0.075***	0.213
Small	Complex	Small	0.816	0.820	***0.940***	0.102	0.141	***0.064***
Small	Complex	Large	0.810	0.811	***0.939***	0.158	0.158	***0.019***
Medium	Simple	No	0.636	***0.944***	0.495	0.183	***0.062***	0.201
Medium	Simple	Small	0.935	0.935	***0.991***	0.079	0.079	***0.014***
Medium	Simple	Large	0.922	0.899	***0.954***	0.089	0.095	***0.011***
Medium	Complex	No	0.585	***0.856***	0.571	0.214	***0.158***	0.195
Medium	Complex	Small	0.805	0.830	***0.950***	0.117	0.111	***0.053***
Medium	Complex	Large	0.800	0.805	***0.940***	0.139	0.112	***0.057***

*Note:* Values with the highest AUC and lowest Brier scores for each level of simulation conditions are in bold and italics.

### 4.2 Simulation study 2

#### 4.2.1 Simulation designs and expected results

Outcomes were generated under the EIRM-tree (i.e., EIRM-RF with a single “true” tree structure) to evaluate interpretable ML methods based on the results of 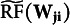 from EIRM-RF. The tree structure of the EIRM-tree was generated using two randomly selected categorical item-level predictors from three item-level predictors and five randomly selected person-level predictors from 13 person-level predictors (four categorical and nine continuous predictors, as used in Simulation Study 1). This generation process means that the values of (normalized) importance measures for unselected predictors are close to 0 in the predicted values of the true tree structure. As in Simulation Study 1, two or three categorical person- or item-level predictors were generated.

It has been shown that importance measures in RF are affected by predictor correlations (e.g., Gregorutti et al., 2017). Therefore, when generating a true tree structure with simulated predictors, the median of small and moderate correlations between predictors—set at 0.003 (a small level, as in the empirical study) and 0.400 (a medium level), respectively,—was considered to evaluate the effects of predictor correlations on the performance of interpretable ML methods. The interquartile range of correlations between predictors was 0.070 for both the small and moderate correlation conditions, as found in the empirical study. Because predictors can vary depending on the correlations among them and the sample sizes, four true tree structures were considered with the sample sizes considered in Simulation Study 1: 0.1 versus 0.4 predictor correlations with 10 items and 100 persons, and 0.1 vs. 0.4 predictor correlations with 30 items and 400 persons. For each true tree structure of four true tree structures, small versus large variances of random effects were examined, as used in Simulation Study 1. The same generated data sets were fit using both EIRM-RF and RF models with the generated item- and person-level predictors. In total, eight simulation conditions were evaluated for the two models, with 1,000 replications conducted for each condition. Two true generated tree structures with split points (0.1 versus 0.4 predictor correlations) using 30 items and 400 persons are presented in Appendix A for illustrative purposes. In addition, the “true” interpretable ML results are reported in Appendix A for illustration. All “true” values are available upon request from the first author of this article. Using 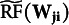 from the true generated tree structures, person-by-item binary outcomes were generated with 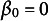 and 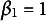 under the EIRM-tree.

To assess interpretable ML methods, the values derived from these methods based on the true tree structure using all 16 predictors were considered the “true” values. As an evaluation measure, we used Spearman’s rank correlation between the true values of the Gini-based importance measures and the corresponding values from the RF component of EIRM-RF and RF, as the interpretation is based on rank order. For simplicity, we refer to this as correlation below. For partial dependence and accumulated local effect plots, the values of partial dependence and accumulated local effects (presented on the *y*-axis in the plots) were calculated for both 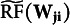 from the generated tree structure (representing the true values) and 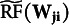 from EIRM-RF (representing the empirical values), using the same values for each predictor (presented on the *x*-axis in the plots). Average correlations between the true values and the empirical values across 1,000 replications were then calculated for the seven selected predictors used in generating the true tree structure.

The following simulation patterns are expected based on prior findings on interpretable ML methods derived from the RF component of EIRM-RF. Variable importance becomes unstable when predictor correlations are high because such correlations make it difficult to discern each predictor’s true contribution (e.g., Gregorutti et al., 2017; Strobl et al., 2007). Consequently, higher correlations between true and empirical measures are anticipated when there are small correlations between predictors, compared to when there are moderate correlations between predictors. A larger sample size may mitigate the effects of predictor correlations by providing more data for RFs to discern subtle differences in their contributions. Smaller random effects may reduce the impact of random variability on the splitting criteria in RFs, thereby enhancing the performance of interpretable ML methods. In addition, feature importance summarizes a predictor’s aggregate influence across the entire dataset and model structure, while partial dependence focuses on depicting the shape and direction of the predictor’s marginal effect on the outcome. However, partial dependence and feature importance rankings may align under certain simulation conditions, as both assess a predictor’s contributions and interactions. For example, when correlations are minimal and sample sizes are adequate, both measures may rank predictors similarly. Similarly, accumulated local effects often yield patterns that resemble the *H*-statistic because both methods rely on characterizing predictor interactions. However, accumulated local effect plots smooth local contributions, which can result in slightly reduced values (2019). In addition, the performance of the interpretable ML methods derived from the RF component of EIRM-RF is expected to be better than that of RF models using only person- and item-level predictors when the data-generating model is an EIRM-tree. This is because the crossed random effects present in the EIRM-tree are ignored by standard RF. Larger differences in correlations between EIRM-RF and RF are expected under the higher variance condition (2.8) than under the lower variance condition (0.4).

#### 4.2.2 Analysis

A 5-repeated 10-fold cross-validation procedure was used to determine the optimal 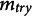 and the number of trees for calculating interpretable ML methods and 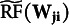 from each of the four true generated tree structures. In addition, the same cross-validation procedure was applied to train the RF component of EIRM-RF, using 10% of replications for each simulation condition. In the grid search, 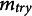 values of 2, 3, 4, and 5 were tested, along with 1000, 1500, 2000, 2500, and 3000 trees. The combination of the two tuning parameters that resulted in the smallest AUC and Brier scores was selected for each simulation condition. In all simulation conditions for EIRM-RF, the optimal model was identified with 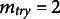 . However, the number of trees varied between 1,000 and 2,000 across simulation conditions. Convergence was reached in fewer than 35 iterations when fitting EIRM-RF. The model accuracy of EIRM-RF is presented in Appendix B. For RF, the grid search considered the same values for 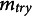 and the number of trees as those used in EIRM-RF. The optimal values of the two tuning parameters ranged from 2 to 4 for 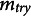 and from 2000 to 3000 for the number of trees across simulation conditions.

#### 4.2.3 Results

In Table 5, the average correlation across the 16 predictors is reported for each condition and for the interpretable ML methods under EIRM-RF and RF, respectively. In line with the anticipated patterns for EIRM-RF, the simulation results generally show that lower predictor correlations, larger sample sizes, and smaller random effects variances yield higher correlations between the true and empirical values of the interpretable ML methods. Under conditions where predictor correlations are small (0.003), all four interpretable ML methods demonstrate relatively high and consistent correlations with the true values. These high correlations become even more pronounced with larger sample sizes and smaller variances, suggesting that when predictors are less confounded and more data are available, it is easier for the RF component of EIRM-RF to recover and correctly rank the true structure.

**Table 5:** Simulation study 2: performance of interpretable ML methods under EIRM-tree.

Conditions			Results from EIRM-RF				Results from RF			
Corr	Sample size	Variance	IM	PD	ALE	*H*-statistic	IM	PD	ALE	*H*-statistic
0.003	Small	0.7	0.908	0.900	0.897	0.899	0.822	0.821	0.803	0.793
0.003	Small	2.8	0.851	0.850	0.880	0.884	0.679	0.690	0.709	0.704
0.003	Medium	0.7	0.962	0.950	0.983	0.986	0.899	0.874	0.902	0.893
0.003	Medium	2.8	0.868	0.870	0.890	0.893	0.721	0.712	0.746	0.713
0.400	Small	0.7	0.641	0.600	0.765	0.769	0.439	0.400	0.568	0.567
0.400	Small	2.8	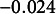	0.000	0.198	0.200	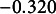	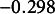	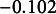	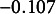
0.400	Medium	0.7	0.930	0.920	0.825	0.828	0.606	0.783	0.685	0.690
0.400	Medium	2.8	0.035	0.035	0.210	0.212	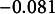	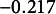	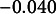	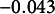

*Note:* IM refers to the Gini-based importance measure; PD refers to the partial dependence effect; and ALE refers to the accumulated local effect.

For importance and partial dependence measures from EIRM-RF, under low predictor-correlation conditions (Corr = 0.003), both methods consistently produce high correlations with the true values, and their correlations are often close to each other. In contrast, when the predictor correlation is moderate (Corr = 0.4), the agreement between the two measures diminishes, particularly under smaller sample sizes and larger variances. Under conditions of Corr = 0.4, smaller sample size, and smaller variance, the correlation drops (importance measure = 0.641, partial dependence = 0.600). Under Corr = 0.4, smaller sample size, and larger variance, the results are even more striking (importance measure = 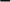 0.024, partial dependence = 0.000), indicating that both measures fail to recover the true structure under these harsher conditions. In this finding, there is no systematic tendency for numerical predictors to exhibit higher importance measures than categorical predictors.

Regarding accumulated local effects and the *H*-statistic from EIRM-RF, the results in Table 5 demonstrate that these two measures remain closely aligned across conditions. For instance, under Corr = 0.003, smaller sample size, and smaller variance, correlations between the true and empirical values are nearly identical (accumulated local effects = 0.897, *H*-statistic = 0.899). Even under less ideal conditions—such as Corr = 0.4, larger sample size, and smaller variance—accumulated local effects (0.825) and the *H*-statistic (0.828) remain in close agreement. Notably, accumulated local effects are consistently slightly lower than the *H*-statistic values, reflecting accumulated local effects’ smoothing of local contributions and resulting in marginally reduced correlation values. This consistent pattern, in which accumulated local effects yield slightly lower but still similar values compared to the *H*-statistic, directly supports the theoretical expectations (Molnar, 2019).

In summary, for EIRM-RF, when predictor correlations are small, sample sizes are larger, and random effects variances are smaller, all interpretable ML methods demonstrate high and consistent correlations with the true values, with importance and partial dependence measures closely aligned and accumulated local effects closely mirroring the *H*-statistic. Under these favorable conditions, high correlations indicate that the interpretable ML methods successfully recover the underlying data-generating structure. Specifically, importance measures reliably identify relevant predictors, *H*-statistics accurately detect interaction effects, and partial dependence and accumulated local effect plots effectively capture nonlinear relationships. However, as predictor correlation increases, sample sizes become smaller, and random variance is large, interpretable ML methods — especially importance and partial dependence measures — fail to recover the true structure.

For the interpretable ML methods derived from RF, similar patterns across simulation conditions were observed, as described above for EIRM-RF. However, overall, the correlations of the interpretable ML methods derived from the RF component of EIRM-RF were higher than those of RF models using only person- and item-level predictors. Overall, this pattern was more pronounced under the higher variance condition than under the lower variance condition, with the exception of the importance measure under the medium sample size and high correlation condition. These results suggest that interpretable methods derived from RF are more biased in the presence of crossed random effects than those derived from the RF component of EIRM-RF.

## 5 Summary and discussion

This study is motivated by the goal of maximally explaining variability in person-by-item binary responses through the use of person- and item-level predictors. This article presents a hybrid approach termed EIRM-RF, and its parameter estimation method using the bimm package with modifications. In EIRM-RF, RF is used to model nonlinear and interacted fixed effects, and simultaneously, random effects across persons and items are considered, as typically done in EIRM. In addition, interpretable ML methods were applied to the results of RF in EIRM-RF to probe the effects of the predictors. The EIRM-RF was demonstrated using an empirical data set to explain differences in reading comprehension in paper and digital mediums. In the empirical study, the model accuracy of EIRM-RF, coupled with interpretable ML methods and ICCs, indicates the presence of nonlinear and interaction fixed effects of person- and item-level predictors, and non-negligible random effects. In addition, the simulation study showed that the model accuracy of EIRM-RF is superior to that of EIRM and RF under conditions of complex fixed-effect structures and the large variances. This result underscores the importance of modeling random effects to leverage RF in the modeling of complex fixed effects of person- and item-level predictors. This finding aligns with those of previous studies (Hajjem et al., 2014; Ngufor et al., 2019; Speiser et al., 2019). However, RF outperformed EIRM and EIRM-RF in no-variance conditions, which might be rare in empirical studies. Furthermore, an additional simulation study showed that all interpretable ML methods based on the RF component of EIRM-RF demonstrated high and consistent correlations with the true values when predictor correlations were small, sample sizes were larger, and random effects variances were smaller. The accuracy of the interpretable ML methods was acceptable ( 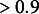 ) under the simulation condition (predictor correlation = 0.003 and random effects variance = 0.7), which is similar to our empirical study.

Given the results of the empirical and simulation studies, we recommend fitting EIRM, RF, and EIRM-RF to the same empirical data set when explaining variability in person-by-item binary responses using person- and item-level predictors. The differences in model accuracy, measured by AUC and Brier Score, between EIRM-RF and EIRM with linear and no-interaction effects could indicate the presence of nonlinear or interacted fixed-effects of person- and item-level predictors. In addition, the differences in model accuracy between EIRM-RF and RF could imply the presence of non-negligible random effects. When notable differences are found between EIRM-RF and RF (with person- and item-level predictors), and between EIRM-RF and EIRM, the results of EIRM-RF and interpretable ML methods can be used to probe complex patterns in item response data using person- and item-level predictors. While EIRM provides point estimates and statistical inferences for the effects of predictors, essential for hypothesis testing and understanding variable impacts, the EIRM-RF focuses on identifying patterns and generating hypotheses regarding predictors. This makes the EIRM-RF particularly useful in studies dealing with complex data structures that have many predictors where the EIRM might falter due to their inability to handle complex nonlinear and interaction relationships among the predictors effectively. However, as predictor correlations increase, sample sizes decrease, and random variance becomes large, interpretable ML methods—particularly importance measures and partial dependence plots—struggle to recover the true underlying structure.

### 5.1 What did we learn from the empirical study?

The results obtained from EIRM-RF provide practical implications that are not attainable through EIRM or RF alone. Together, EIRM-RF allowed us to look beyond what features mattered to consider how they matter. Specifically, the results of the empirical study showed how these features together linked to reading comprehension identifying aspects like which play a particularly important role, which are best interpreted in nuanced ways that allow for nonlinear relations, and which need to be considered in concert with other features such that certain behaviors may be particularly dependent on other behaviors. Recently, reviews of digital reading have called for careful consideration of digital reading processes (Singer & Alexander, 2017) that attend to the complexities of reading and especially digital reading (Coiro, 2021). To do this, researchers suggest moving beyond modeling the role of a single feature to instead model how multiple features together contribute to reading comprehension under different conditions. In other words, when considering digital reading, researchers should look at the role of highlighting specifically but then also in concert with other features like annotating and looking back and then considering context like item task and format because these features often interact in important ways. For example, a reader might be able to look back more effectively if the reader had highlighted and annotated the information in the question being assessed.

Overall, the results of the empirical study confirm much of what is in the reading literature such as the importance of considering item format and task when modeling reading comprehension. Here, the current study showed interaction effects strongly contribute to understanding reading comprehension (i.e., via the *H* statistic). This is not surprising given the often cited RAND reading report that emphasizes that reading comprehension depends on reader, text, and task characteristics (Snow, 2002), but it shows which variables are interacting in important, quantifiable ways. Researchers can consider these more complicated relationships in further modeling to get the most accurate understandings to inform research, theory, and practice. For example, item format was shown to interact with a large number of predictors. Modeling of the interactions related to item format showed that format interacted with a variety of variables especially part, task, white (race), looking back on paper, grade, looking back digitally, and number of paper highlights. The next step would be to explore these interactions further to see, for example, how looking back (on paper or digital) or highlighting made certain question formats (i.e., multiple choice versus true/false) easier or harder. Another example might involve exploring how the format of the question interacts with the task, such as multiple-choice questions being particularly challenging for inferencing questions compared to locate-and-recall questions, where the answer is present in the text. The point here is that identification of interactions provides insights that deepen understandings and also lead to important future explorations. We see examples from this in the larger reading literature. Theories like New Literacies (Leu et al., 2013) call out exploration of different technologies in a nuanced way, to which this work adds additional variable to consider. Similarly, studies like Ben-Yehudah & Eshet-Alkalai (2018) provide further evidence for modeling interactions as they showed the effect of highlighting on comprehension was positive only for paper reading and specifically for questions about higher-level inferencing and processing (not lower-level factual recall), which shows the importance of task. Our study suggests that understanding interactions can provide important guidance to assessment and instruction such that curriculum designers and teachers should be wary of statements that indicate using the same behaviors for all questions. Rather than directing students to, for example, highlight five things, educators might encourage students to be strategic about responding to questions of certain task and format types.

In addition, this study deepens understandings of reading comprehension in different mediums by showing important nuances. As in EIRM with linear effects, EIRM-RF results show that looking back on paper and total response time are significant predictors. However, the relationship between these reading behaviors and outcome (reading comprehension) were not linear in EIRM-RF. For example, response time was shown to be important for those who were relatively quick, and again less so for those who took longer times. This indicates that readers should take some time to think about answers, and possibly check those answers by looking back, given that they already have a strong mental model of the text that supports this timely consideration. In contrast, spending large amounts of time looking back or even fully re-reading the text to create a mental model if a weak one was created initially is less supportive of accurate comprehension. Using the initial, unprepared read to develop a strong mental model can be leveraged to support timely and thoughtful evaluation of answers, as opposed to making hasty guesses or responding impulsively. This approach seems to be an efficient means of comprehension. In another example for both digital and paper looking back, the results of the current empirical study indicated that there seems to be a threshold for which looking back is particularly helpful but also after which additional look backs do not add, which adds a layer to thoughtful answering. It is likely that building meta-cognitive awareness of looking back as a support for a strong mental model of comprehension would be an effective strategy for readers.

These nuances are important generally and fit within calls to deepen understanding of how behaviors work together in different contexts (e.g., Leu et al., 2013). In particular, when aiming to understand digital and paper reading, the current study was able to show important differences between how digital and paper behaviors relate to comprehension. EIRM as used in past work (Goodwin et al., 2020) showed digital highlighting significantly predicting comprehension whereas paper highlighting did not. Yet our work unraveled this further, showing digital highlighting supported comprehension up to a point likely emphasizing the importance of strategic highlighting as fewer highlights likely draws attention to important ideas whereas prolific highlighting makes it hard to discern important ideas from less important ideas. In terms of relations to other features, we found that paper highlighting and looking back were more related to other features than their digital counterparts. This may indicate that readers are more able to apply these behaviors in concert with others and adapt them to contexts whereas the digital behaviors, perhaps which are new given many of the classrooms in this study reported having more paper reading activities, may be less reliant on other features, perhaps indicating less concerted use towards comprehension although future research should look at this in greater detail.

### 5.2 Methodological limitations of the current study and future study

In the model specification of EIRM-RF (Equation 5), the fixed effect ( 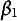 ) of the predicted RF values (which vary across persons and items) is considered, assuming it does not vary across persons and items. As a more flexible approach, random slopes of the predicted RF values can be introduced to model variability in these predicted values across persons and items, which would involve cross-classified random slopes.^4^ This flexible modeling approach requires further development and evaluation of the presented estimation methods to estimate the cross-classified random slopes, which is beyond the scope of the current study. Once the estimation method for random slopes of the predicted RF values is available, it would be advisable to compare the evaluation measures and interpretable methods of EIRM-RF with and without the random slopes.

Furthermore, the predicted values ( 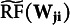 ) in EIRM-RF are on the probability scale. When predicted values are near 0 or 1, their variability is limited due to the bounded nature of the probability scale, reducing the model’s sensitivity to detect subtle effects. This constraint can diminish the explanatory power, especially when used in models on the logit scale.^5^ Alternative transformation or calibration methods can be considered. For example, applying a logit transformation to 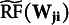 would convert these probability estimates into the log-odds scale, which ranges from minus infinity to infinity. Similarly, calibration techniques—such as isotonic regression (Zadrozny & Elkan, 2002) or Platt scaling (Platt, 1999), which are often used to adjust the outputs of ensemble methods—could also be employed to harmonize the predictor scales. Investigating these approaches is a promising avenue for future research, potentially leading to improved model performance and interpretability.

If the goal of using EIRM-RF is to explain variability in item responses within a study as in the current study using the bootstrap samples (thereby reducing the variance) for RF in EIRM-RF, rather than predicting unseen data or generalizing empirical findings to other similar study designs, the necessity of splitting data into training and test data sets becomes less critical. In explanatory modeling, the focus is primarily on understanding the relationships between predictors and outcome variables, and identifying key predictors. While data splitting is less critical in explanatory modeling compared to predictive modeling (for unseen data), we recommend data splitting if the objective of EIRM-RF applications is to predict unseen data or to generalize empirical findings, especially when there are a large number of persons and items.

When EIRM-RF is applied to cross-classified person-by-item binary item responses with data splitting, the unit of sampling can become more complex compared to nested data (e.g., students nested within schools). In nested data, it is common practice to split the data into training and test data sets by clusters (e.g., Ngufor et al., 2019; Speiser et al., 2019). However, with cross-classified person-by-item binary item responses, where clusters comprise both persons and items, it is necessary to decide how to select clusters for data splitting. In the context of explanatory item response modeling, the primary goal is to explain individual differences in a latent variable as measured by a set of items. Therefore, when a large number of persons is available, we recommend that, in data splitting, clusters could be formed based on persons rather than on items, or a combination of both. Nevertheless, future studies are needed to discuss and assess various methods of data splitting in cross-classified data sets.

In addition, for predictive models, predictions are made for clusters in multilevel data using both fixed and random effects (residuals) in the training data set. However, in the testing data, predictions are made using only the fixed effects because the random effects are not known (e.g., Ngufor et al., 2019). In contrast, in explanatory models like those in the current study, both fixed and random effects should be used in prediction.

Furthermore, in practical applications, it is not uncommon to encounter new datasets with additional predictors, missing predictors, or even different distributions of the same predictors. While our model specification is not specifically designed for prediction in such scenarios, it can still be useful as a baseline model for adaptations. Practitioners should analyze the comparability of predictors between datasets, and if discrepancies are significant, further refinement or alternative models may be needed for accurate predictions. Future research could further explore these aspects, particularly in the context of developing more robust models for generalization across different datasets.

The simulation studies in the current research were designed to highlight the differences in model accuracy among EIRM, RF, and EIRM-RF with respect to varying fixed-effect structures, degrees of variance, and sample sizes, as well as to evaluate interpretable ML methods under limited conditions. Thus, the simulation results should be interpreted within the context of the selected simulation conditions. As an inherent limitation of the simulation study, additional simulations are required to generalize the findings to other scenarios not considered in this study. As an example of the additional simulation condition, a multidimensional structure of random effects can be considered in data generation as used in Park et al. (2023) to investigate the behaviors of RF and EIRM-RF in the presence of multidimensionality. In addition, the simulation study 1 showed robust performance of the two-step procedure. However, formal proofs regarding its convergence and consistency have not been provided, leaving this as an open area for future research.

Strobl et al. (2007) demonstrated that variable selection bias can occur in tree-based methods, particularly when using the Gini impurity measure, which may favor predictors with a larger number of categories or those measured on a larger scale. In the second simulation study, however, there is no systematic tendency for continuous predictors to exhibit higher importance measures than categorical predictors, based on the predicted values of RF from EIRM-RF. It is important to note that the simulation was conducted under specific conditions—comprising 13 person-level predictors (four categorical and nine continuous) and three categorical item-level predictors, as found in the empirical study—and therefore the findings may not generalize to all scenarios or datasets. A systematic investigation into the differential behavior of various importance measures (Gini importance versus permutation importance) for categorical versus continuous variables, using different approaches (e.g., marginal versus conditional approach), would require a dedicated study for EIRM-RF, similar to the approach taken by Strobl et al. (2007).

### 5.3 Conclusions and extensions of the current study

Despite the methodological limitations, the present study demonstrates the applicability of incorporating RF into an item response model, and using interpretable ML methods to probe complex nonlinear and interactions of person- and item-level predictors. As the first step in this effort, the study considered a cross-sectional (single-level) person-by-item data set. The EIRM-RF can be extended to other types of data, such as cross-sectional multilevel data (e.g., students nested within schools) and longitudinal person-by-item response data, by adapting different random effect structures in EIRM-RF to account for correlations in such data. In addition, we considered RF among supervised ML methods in the current study, based on prior research (e.g., Park et al., 2023). However, any supervised ML methods can be incorporated into an item response model as EIRM, for the purpose of explaining or predicting item responses using person- and item-level predictors. We hope that this study will serve as an initial effort towards these extensions in the application of EIRM.

## Figures and Tables

**Figure A.1:**
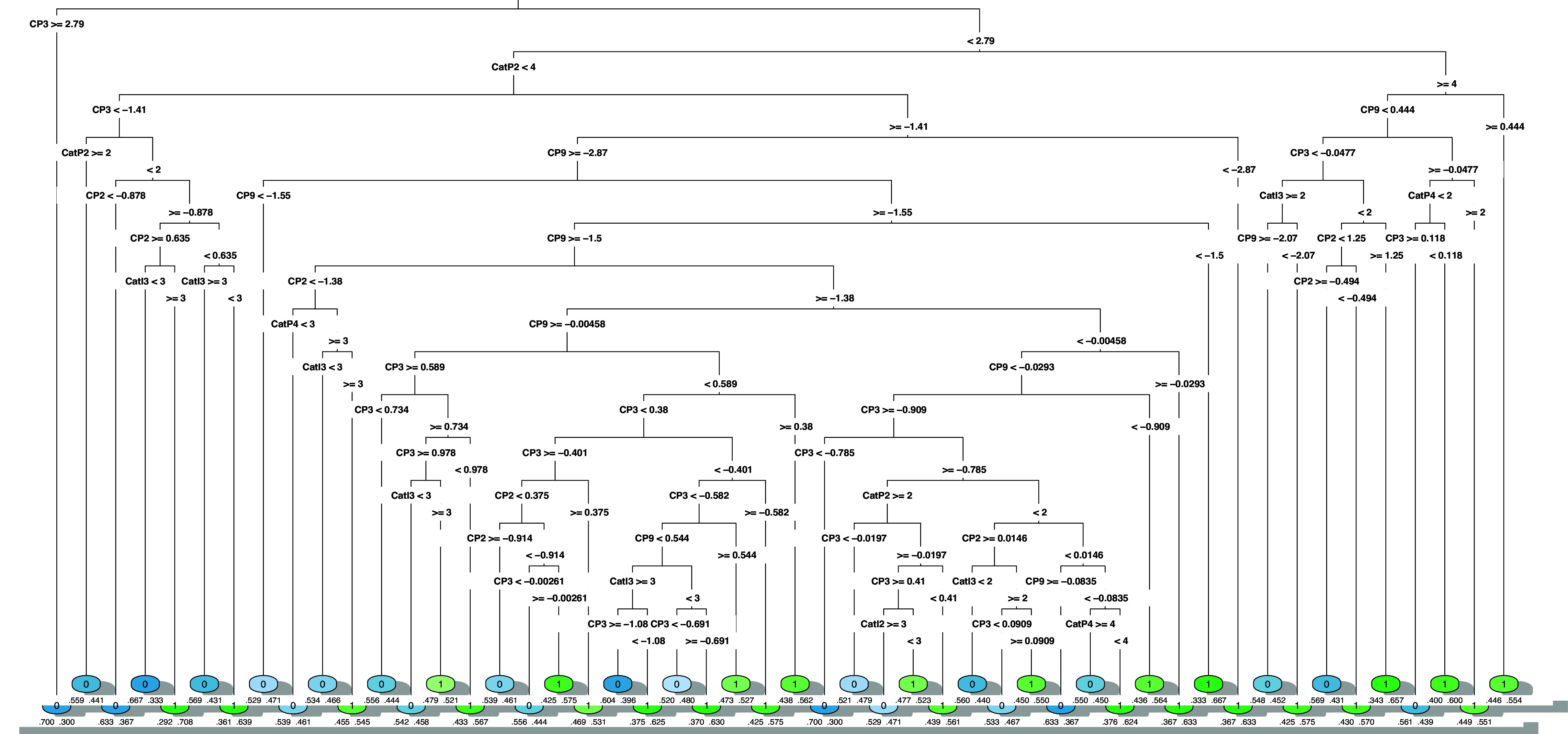
Visualization of a generated tree structure.

**Figure A2:**
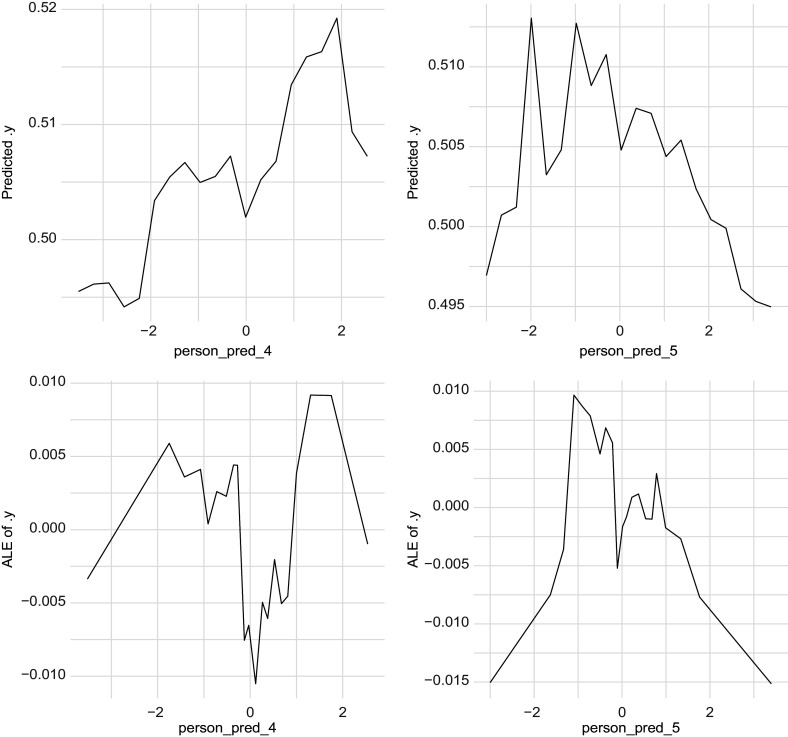
Selected plots of ‘true’ partial dependence (top) and accumulated local effects (bottom). *Note*: Continuous predictors were mean-centered; To present patterns in partial dependence and accumulated local effects, the *x*- and *y*-axes of the two selected predictors were not displayed on the same scale.

**Figure A3:**
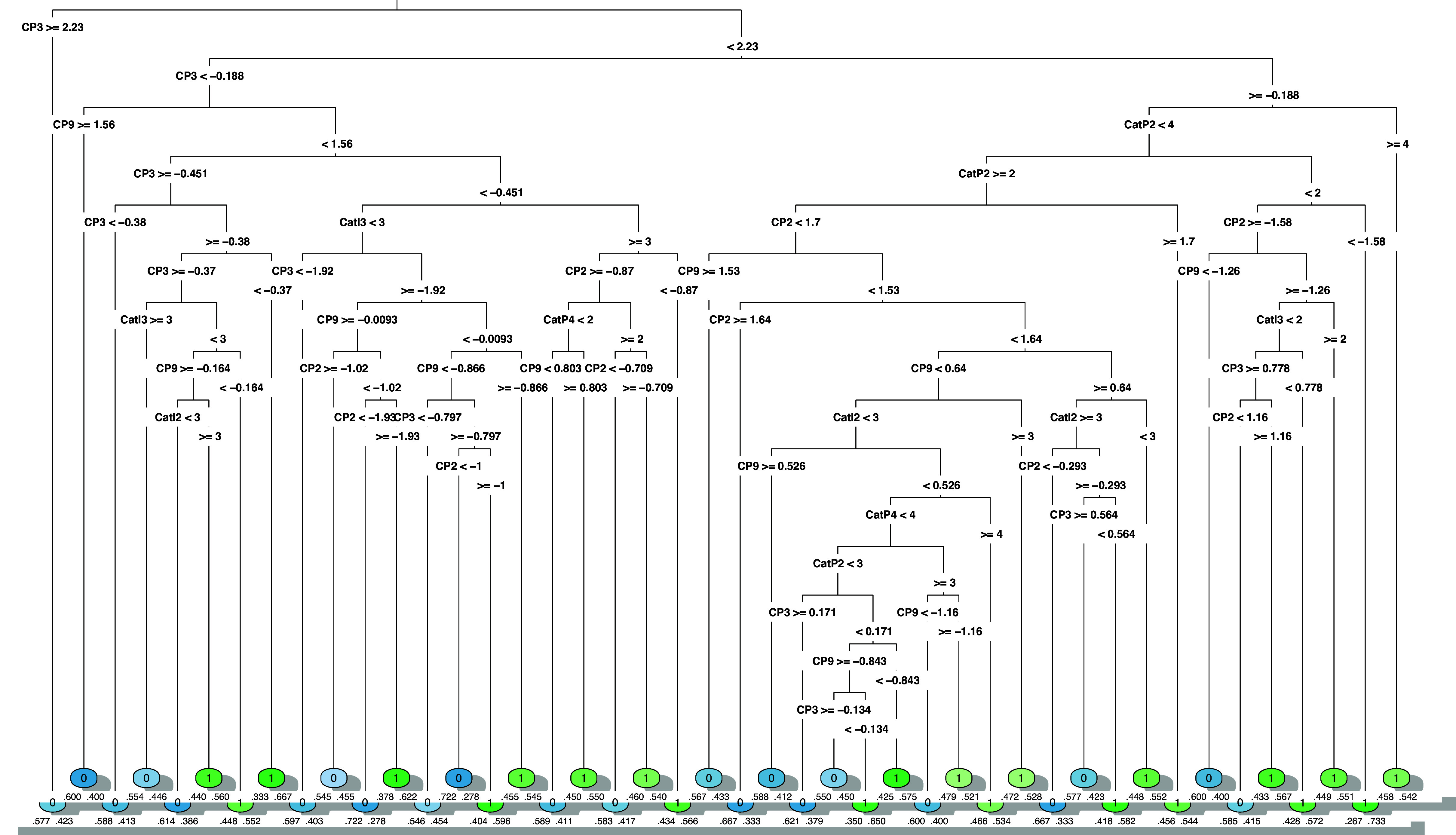
Visualization of a generated tree structure.

**Figure A4:**
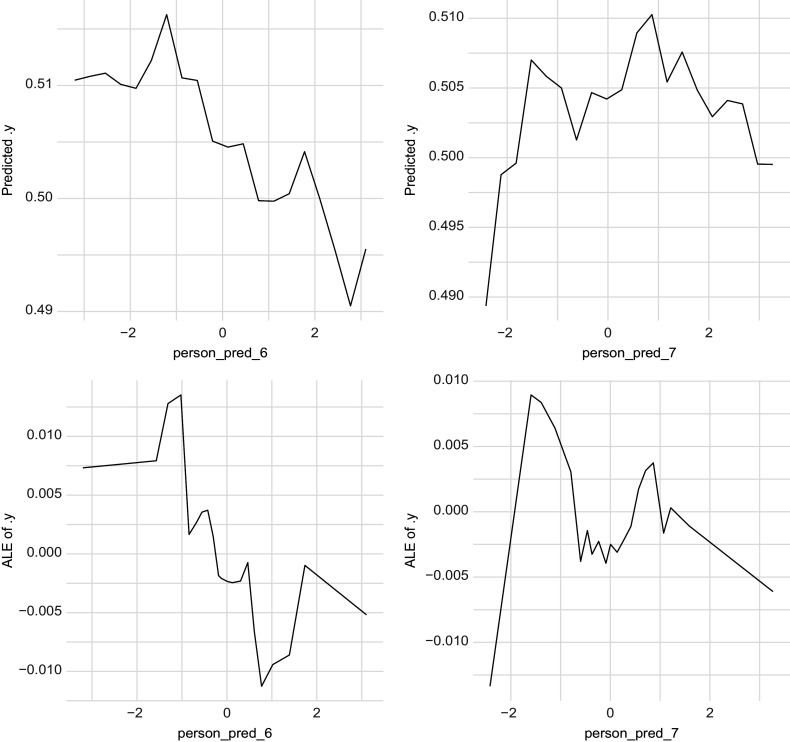
Selected plots of “true” partial dependence (top) and accumulated local effects (bottom). *Note*: Continuous predictors were mean-centered; To present patterns in partial dependence and accumulated local effects, the *x*- and *y*-axes of the two selected predictors were not displayed on the same scale.

## Data Availability

The data and R code used in the illustration are available on the Open Science Framework at https://osf.io/dbjpu/.

## Footnotes

1 In this study, the terms “predictors” and “features” are used interchangeably.

2 The term “model adequacy” refers to the degree to which a statistical model accurately represents the data it is intended to describe.

3 The term “small” does not refer to an absolute size of residual variances. In practice, it may represent a medium size of variances. Both small and large variances should be interpreted relatively, rather than absolutely, within the context of a simulation condition.

4 We thank an anonymous reviewer for raising this point.

5 We acknowledge an anonymous reviewer for suggesting this clarification.

## Appendices

### A Simulation study 2: visualization of generated tree structures and true interpretable ML methods for the selected two conditions

**Condition of 0.1 predictor correlation using 30 items and 400 persons**

**Table A1:** Normalized importance measure and *H*-statistic for each predictor.

Predictor	Normalized importance	-statistic
item_pred_1	0.1452	0.4870
item_pred_2	0.0041	0.0125
item_pred_3	0.1386	0.4532
person_pred_1	0.0057	0.0213
person_pred_2	0.1204	0.3765
person_pred_3	0.0061	0.0184
person_pred_4	0.0983	0.3281
person_pred_5	0.0038	0.0112
person_pred_6	0.0044	0.0175
person_pred_7	0.1032	0.3397
person_pred_8	0.0029	0.0108
person_pred_9	0.0035	0.0129
person_pred_10	0.1110	0.3620
person_pred_11	0.0048	0.0152
person_pred_12	0.1195	0.3914
person_pred_13	0.0037	0.0139

**Condition of 0.4 predictor correlation using 30 items and 400 persons**

**Table A2:** Normalized importance measure and *H*-statistic for each predictor.

Predictor	Normalized importance	-statistic
item_pred_1	0.1431	0.4720
item_pred_2	0.0055	0.0132
item_pred_3	0.1357	0.4385
person_pred_1	0.0054	0.0115
person_pred_2	0.1441	0.3680
person_pred_3	0.0045	0.0091
person_pred_4	0.1389	0.3179
person_pred_5	0.0045	0.0102
person_pred_6	0.0044	0.0147
person_pred_7	0.1337	0.3456
person_pred_8	0.0040	0.0104
person_pred_9	0.0039	0.0118
person_pred_10	0.1337	0.3550
person_pred_11	0.0039	0.0140
person_pred_12	0.1316	0.3862
person_pred_13	0.0034	0.0122

### B Simulation study 2: model accuracy of EIRM-RF

**Table B1:** Model accuracy of EIRM-RF

	Conditions		Model accuracy	
Corr	Sample size	Variance	AUC	Brier score
0.003	Small	0.7	0.942	0.061
0.003	Small	2.8	0.933	0.046
0.003	Medium	0.7	0.970	0.051
0.003	Medium	2.8	0.955	0.059
0.400	Small	0.7	0.720	0.178
0.400	Small	2.8	0.695	0.188
0.400	Medium	0.7	0.790	0.150
0.400	Medium	2.8	0.765	0.167

## References

[r1] Apley, D. W. , & Zhu, J. (2020). Visualizing the effects of predictor variables in black box supervised learning models. Journal of the Royal Statistical Society: Series B (Statistical Methodology), 82, 1059–1086. 10.1111/rssb.12377

[r2] Baayen, R. H. , Davidson, D. J. , & Bates, D. M. (2008). Mixed-effects modeling with crossed random effects for subjects and items. Journal of Memory and Language, 59, 390–412. 10.1016/j.jml.2007.12.005

[r3] Bates, D. , Mächler, M. , Bolker, B. , & Walker, S. (2015). Fitting linear mixed-effects models Using lme4. Journal of Statistical Software, 67, 1–48. 10.18637/jss.v067.i01.

[r4] Ben-Yehudah, G. , & Eshet-Alkalai, Y. (2018). The contribution of text-highlighting to comprehension: A comparison of print and digital reading. Journal of Educational Multimedia and Hypermedia, 27, 153–178.

[r5] Bolsinova, M. , & Molenaar, D. (2018). Modeling nonlinear conditional dependence between response time and accuracy. Frontiers in Psychology, 9, 12. 10.3389/fpsyg.2018.01525 30245650 PMC6137682

[r6] Breiman, L. , Friedman, J. H. , Olshen, R. , & Stone, C. J. (1984). Classification and regression trees. Wadsworth & Brooks/Cole.

[r7] Breiman, L. (1996). Bagging predictors. Machine Learning, 24, 123–140. 10.1007/BF00058655

[r8] Breiman, L. (2001). Random forests. Machine Learning, 45, 5–32. 10.1023/A:1010933404324

[r9] Cho, S.-J. , & Rabe-Hesketh, S. (2011). Alternating imputation posterior estimation of models with crossed random effects. Computational Statistics and Data Analysis, 55, 12–25. 10.1016/j.csda.2010.04.015

[r10] Cho, S.-J. , Partchev, I. , & De Boeck, P. (2012). Parameter estimation of multiple item response profile model. British Journal of Mathematical and Statistical Psychology, 65, 438–466. 10.1111/j.2044-8317.2011.02036.x 22070786

[r11] Coiro, J. (2021). Toward a multifaceted heuristic of digital reading to inform assessment, research, practice, and policy. Reading Research Quarterly, 56, 9–31. 10.1002/rrq.302

[r12] De Boeck, P. , & Wilson, M. (2004). Explanatory item response models: A generalized linear and nonlinear approach. Springer. 10.1007/978-1-4757-3990-9_1

[r13] De Boeck, P. (2008). Random item IRT models. Psychometrika, 73, 533–559. 10.1007/s11336-008-9092-x

[r14] Friedman, J. H. (2001). Greedy function approximation: A gradient boosting machine. Annals of Statistics, 29, 1189–1232. 10.1214/aos/1013203451

[r15] Friedman, J. H. , & Popescu, B. E. (2008). Predictive learning via rule ensembles. The Annals of Applied Statistics, 2, 916–954.

[r16] Goodwin, A. P. , Cho, S.-J. , Reynolds, D. , Brady, K. , & Salas, J. A. (2020). Digital versus paper reading processes and links to comprehension for middle school students. The American Educational Research Journal, 57, 1837–1867. 10.3102/0002831219890300

[r17] Greenwell, B. M. , & Boehmke, B. C. (2020). Variable importance plots—An introduction to the vip package. The R Journal, 12, 343–366. 10.32614/RJ-2020-013.

[r18] Gregorutti, B. , Michel, B. , & Saint-Pierre, P. (2017). Correlation and variable importance in random forests. Statistics and Computing, 27, 659–678. 10.1007/s11222-016-9646-1

[r19] Hajjem, A. , Bellavance, F. , & Larocque, D. (2014). Mixed-effects random forest for clustered data. Journal of Statistical Computation and Simulation, 84, 1313–1328.

[r20] Jacobucci, R. , Grimm, K. J. , & Zhang, Z. (2023). Machine learning for social and behavioral research. Guilford Press.

[r21] Kuhn, M. (2008). Building predictive models in R using the caret package. Journal of Statistical Software, 28, 1–26. 10.18637/jss.v028.i05.27774042

[r22] Kuhn, M. , & Johnson, K. (2018). Applied predictive modeling. (2^nd^ ed.) Springer.

[r23] Leu, D. J. , Kinzer, C. K. , Coiro, J. , Castek, J. , & Henry, L. A. (2013). New literacies: A dual level theory of the changing nature of literacy, instruction, and assessment. In D. E. Alvermann , N. J. Unrau , & R. B. Ruddell (Eds.), Theoretical models and processes of reading (6^th^ ed., pp. 1150–1181). International Reading Association.

[r24] Molnar, C. (2019). Interpretable machine learning. Lulu.com.

[r25] Molnar, C. , Bischl, B. , & Casalicchio, G. (2018). iml: An R package for interpretable machine learning. Journal of Open Source Software, 3, 786. 10.21105/joss.00786.

[r26] Ngufor, C. , Van Houten, H. , Caffo, B. S. , Shah, N. D. , & McCoy, R. G. (2019). Mixed effect machine learning: A framework for predicting longitudinal change in hemoglobin A1c. Journal of Biomedical Informatics, 89, 56–67. 10.1016/j.jbi.2018.09.001 30189255 PMC6495570

[r27] Park, J. Y. , Dedja, K. , Pliakos, K. , Kim, J. , Joo, S. , Cornillie, F. , Vens, C. , & Van den Noortgate, W. (2023). Comparing the prediction performance of item response theory and machine learning methods on item responses for educational assessments. Behavior Research Methods, 55, 2109–2124. 10.3758/s13428-022-01910-8 35819719 PMC9275388

[r28] Platt, J. (1999). Probabilistic outputs for support vector machines and comparisons to regularized likelihood methods. In A. J. Smola , P. Bartlett , B. Schölkopf , & D. Schuurmans (Eds.), Advances in large margin classifiers (pp. 61–74). MIT Press.

[r29] Pliakos, K. , Joo, S. , Park, J. Y. , Cornillie, F. , Vens, C. , & Van den Noortgate, W. (2019). Integrating machine learning into item response theory for addressing the cold start problem in adaptive learning systems. Computers and Education, 137, 91–103.

[r30] R Core Team. (2023). *R: A language and environment for statistical computing*. R Foundation for Statistical Computing. https://www.R-project.org/

[r31] Singer, L. M. , & Alexander, P. A. (2017). Reading on paper and digitally: What the past decades of empirical research reveal. Review of Educational Research, 87, 1007–1041. 10.3102/0034654317722961

[r32] Snow, C. E. (2002). Reading for understanding: Toward an R & D program in reading comprehension. RAND Corporation.

[r33] Speiser, J. L. , Wolf, B. J. , Chung, D. , Karvellas, C. J. , Koch, D. G. , & Durkalski, V. L. (2019). BiMM forest: A random forest method for modeling clustered and longitudinal binary outcomes. Chemometrics and Intelligent Laboratory Systems, 185, 122–134. 10.1016/j.chemolab.2019.01.002 31656362 PMC6813794

[r34] Steacy, L. M. (2020). Capitalizing on the promise of item-level analyses to inform new understandings of word reading development. Annals of Dyslexia, 70, 153–159. 10.1007/s11881-020-00203-z 32666387 PMC8168295

[r35] Strobl, C. , Boulesteix, A.-L. , Zeileis, A. , & Hothorn, T. (2007). Bias in random forest variable importance measures: Illustrations, sources and a solution. BMC Bioinformatics, 8, 25. 10.1186/1471-2105-8-25 17254353 PMC1796903

[r36] Wright, M. N. , & Ziegler, A. (2017). Ranger: A fast implementation of random forests for high dimensional data in C++ and R. Journal of Statistical Software, 77, 1–17. 10.18637/jss.v077.i01

[r37] Zadrozny, B. , & Elkan, C. (2002). Transforming classifier scores into accurate multiclass probability estimates. In Proceedings of the Eighth ACM SIGKDD International Conference on Knowledge Discovery and Data Mining (pp. 694–699). 10.1145/775047.775151

